# A Micromanipulation‐Actuated Large‐Scale Screening to Identify Optimized Microphysiological Model Parameters in Skeletal Muscle Regeneration

**DOI:** 10.1002/advs.202403622

**Published:** 2024-09-12

**Authors:** Xie Chen, Tao Sun, Shingo Shimoda, Huaping Wang, Qiang Huang, Toshio Fukuda, Qing Shi

**Affiliations:** ^1^ Intelligent Robotics Institute School of Mechatronical Engineering Beijing Institute of Technology Beijing 100081 P. R. China; ^2^ Graduate School of Medicine Nagoya University Nagoya 466‐8550 Japan; ^3^ Institute of Innovation for Future Society Nagoya University Nagoya 466‐8550 Japan

**Keywords:** contractile force, large‐scale screening, microphysiological model, skeletal muscle tissue, viscoelasticity

## Abstract

Hydrogel‐based 3D cell cultures are extensively utilized to create biomimetic cellular microstructures. However, there is still lack of effective method for both evaluation of the complex interaction of cells with hydrogel and the functionality of the resulting micro‐structures. This limitation impedes the further application of these microstructures as microphysiological models (microPMs) for the screening of potential culture condition combinations to enhance the skeletal muscle regeneration. This paper introduces a two‐probe micromanipulation method for the large‐scale assessment of viscoelasticity and contractile force (CF) of skeletal muscle microPMs, which are produced in high‐throughput via microfluidic spinning and 96‐well culture. The collected data demonstrate that viscoelasticity parameters (E^*^ and *tanδ*) and CF both measured in a solution environment are indicative of the formation of cellular structures without hydrogel residue and the subsequent generation of myotubes, respectively. This study have developed screening criterias that integrate E^*^, *tanδ*, and CF to examine the effects of multifactorial interactions on muscle fiber repair under hypoxic conditions and within bioprinted bipennate muscle structures. This approach has improved the quality of hypoxic threshold evaluation and aligned cell growth in 3D. The proposed method is useful in exploring the role of different factors in muscle tissue regeneration with limited resources.

## Graphical abstract

A two‐probe micromanipulation method for the large‐scale assessment of viscoelasticity and contractile force (CF) of fiber‐shaped skeletal muscle micro physiological models. The results demonstrate that viscoelasticity parameters (*E* ^*^ and *tanδ*) and CF can be utilized a new combinatorial screening criterion expected to explore the complex interaction of cells with biomaterials for accelerating the development of high‐performance muscle tissue regeneration.

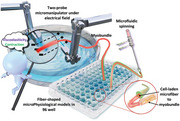

## 1 Introduction

Tissue engineering aims to develop 3D scaffold‐based culture models that accurately represent the physiological and pathological features of native skeletal muscle, in contrast to 2D cultures.^[^ ^1^, ^2^ ^]^ These 3D models have great potential as microphysiological models (microPMs) to replace animal testing in the development of new drug discovery research and regenerative therapies.^[^ ^3^, ^4^ ^]^ Due to complex tissue regeneration process and the low success rate of new drug discovery, microPMs should have the capacity to screen a large‐scale of biochemical and biophysical parameter combinations to address the requirement of a combination of serendipity and many series of trial‐and‐error experiments.^[^ ^5^ ^]^ Conventional screening assessment for microPMs mainly depends on immunofluorescence staining and molecular biological technology.^[^ ^6^ ^]^ These methods can accurately analyze the differentiation status of cultured cells. However, it is difficult to precisely reflect the tissue‐level function based on specific cell organization through individual cell behavior alone.^[^ ^7^, ^8^ ^]^ For instance, in addition to myoblast differentiation and fusion, aligned cell assembly matched with scaffold degradation is another crucial factor for the functional regeneration of engineered skeletal muscle tissue (SMTs).^[^ ^9^ ^]^ Therefore, a screening method specific to muscle tissues has been developed to measure contractile force (CF) for the evaluation of SMT functionality.^[^ ^10^, ^11^, ^12^ ^]^ The method utilizes on‐chip cantilever deflection, post deflection, and force transducers. While the transducers provide high‐precision force measurement, their high cost makes it difficult to significantly expand their number.^[^ ^13^ ^]^ To achieve high‐throughput screening for CF, micro‐cantilevers and posts with a fixed interval distance can be assembled into a 96‐well plate. However, the magnitude of CF is closely related to the initial length of SMT prior to stimulation. It is important to note that a fixed distance between micro‐cantilevers and posts may weaken CF.^[^ ^14^ ^]^ Moreover, the variability of CF induced by the complex process of cell growth with hydrogel degradation may hinder any reliable magnitude comparison between different culturing methods.^[^ ^15^ ^]^ Therefore, it is necessary to develop other new evaluation method for effective and precise screening to promote the regeneration of engineered skeletal muscles.

Over the last two decades, studies have shown that the elasticity or stiffness of the extracellular matrix (ECM) affects fundamental cellular processes.^[^ ^16^ ^]^ Additionally, research has demonstrated a close relationship between the development of neo‐tissue in a degrading scaffold and changes in the structure's stiffness. For instance, when compared to the initial elastic modulus of the cell‐laden structure, the modulus initially decreases to a lower value due to scaffold degradation. However, it subsequently recovers to a higher value due to the coupled reconstitution of the ECM, indicating the chondrogenesis process.^[^ ^17^ ^]^ These studies primarily focus on the stiffness of the matrix, while the resulting tissues and ECMs are complex viscoelastic materials.^[^ ^18^ ^]^ Various techniques have been developed to determine the viscoelastic properties of materials at different length scales, ranging from centimeters to nanometers. These techniques include shear rheology, compression/tensile testing, and probe‐based methods.^[^ ^19^, ^20^ ^]^ It is widely recognized that the viscoelastic properties of the surrounding hydrogel, such as stress relaxation, can affect various activities of encapsulated cells, including cell spreading, cell cycle progression, differentiation, and stem cell stemness.^[^ ^21^, ^22^, ^23^, ^24^ ^]^ Objective evidences also suggests that matrix viscoelasticity can indicate physiological processes, including tissue development and disease progression.^[^ ^25^ ^]^ Viscoelasticity parameters, such as complex modulus (E^*^) and the ratio of loss and storage moduli (*tanδ*), have been used to create maps of viscoelastic properties in various tissues, such as muscle, liver, and brain.^[^ ^26^ ^]^ It is important to note that a map of the viscoelastic properties of normal and diseased tissues can indicate disease progression, such as hepatic liver fibrosis.^[^ ^27^ ^]^ Additionally, viscoelasticity change has been proved to be correlated with the process of scaffold degradation with neo‐tissue regeneration.^[^ ^28^ ^]^ However, whether viscoelasticity can be utilized as a new evaluation indicator for evaluating muscle tissue regeneration remains untested.

**Figure 1:**
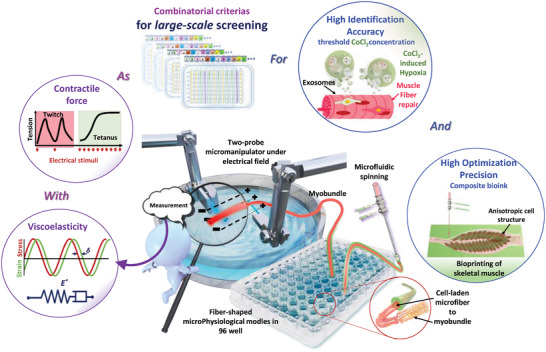
Schematic diagram of the fabrication of fiber‐shaped microPMs, micromanipulator‐actuated measurement for viscoelasticity and CF of myobundles fabricated in microPMs, and then large‐scale screening for identification and optimization of key factors to promote skeletal muscle regeneration based on the criterias combining with viscoelasticity and CF.

In this paper, we achieved the measurement of E^*^, *tanδ*, and CF for same one fiber‐shaped microPM in solution, distinct from the traditional measurement performed in air using a rheomoter, which was used as evaluating parameters to conduct large‐scale screening to identify and optimize key factors in skeletal muscle regeneration in vitro, as illustrated in **Figure** **1**. A meter‐long core‐shell microfiber was spun and divided into 96 wells for the high‐throughput fabrication of myobundles as microPMs. Afterward, the resulting myobundles were easily transferred and placed onto a two‐probe micromanipulator for oscillatory stretching and electrically stimulated active contraction. Real‐time measurements of stress and strain could be calculated to determine E^*^, *tanδ*, and CF of myobundles. The statistical results for over 1400 examples under different biomaterial parameter combinations and culturing conditions indicated that changes in E^*^ and *tanδ* could be used to evaluate the formation of myobundles. The modified gap between two probes allows CF generated micro‐scale sample volumes of myobundles to be clearly detected, enabling the evaluation of their functionality. Based on three screening criterias established by combining with these parameters, large‐scale microPMs screening can accurately identify exosome function for muscle repair and precisely optimize the biomaterial component mixture ratio for skeletal muscle regeneration.

## 2 Results

### 2.1 Design of Micromanipulation Strategy for Fiber‐Based Microphysiological Models

**Figure 2:**
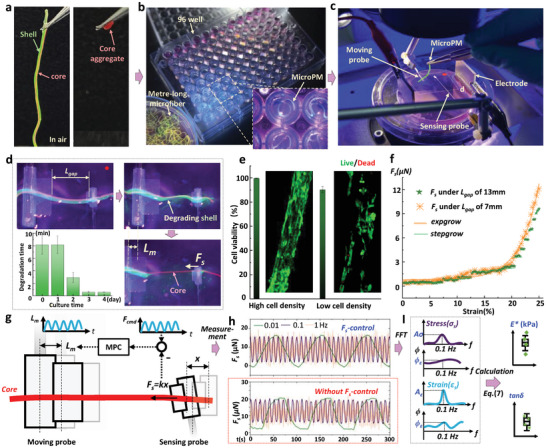
Manipulation strategy for microPMs. a) Shell protection for transferring microPM in air. b) 96 well for high‐throughput culture of microPMs. c) Two‐probe micromanipulator with electrodes for cyclically stretching the core of microPMs. d) Degradation process of the shell. Degradation time recorded from the moment microPM is placed on the two probes until the shell degrades completely, and culture time indicating the duration of culturing microPM in DMEM. Data are expressed as mean ± SD (n =5). e) Verification of cell compatibility of transfer and degradation micromanipulation. f) The relationship between strain and *F_s_* with two *L_gap_*. g) Schematic of stretching manipulation based on MPC. h) Evaluated *F_s_* of microPMs under cyclic loading with or without *F_s_*‐control. (g) Schematic of calculating viscoelasticity parameters.

A metre‐long core‐shell microfiber was formed by encapsulating C2C12 myoblasts‐laden GelMA hydrogel core with an outer Ca‐alginate hydrogel (CA), using a coaxial microfluidic device (Figure S1, Supporting Information). The core and shell were utilized as biochemical and biophysical cue for promoting cell growth and directing cell alignment in 3D, respectively. The Ca‐alginate hydrogel shell had a mechanical strength (MS) of ≈72 kPa and elastic modulus of 24 kPa (as shown in Figure S2, Supporting Information), which provided a protective sheath for the GelMA core (MS: ≈5 kPa, modulus: 3.7 kPa) against significant deformation during manipulation in air.^[^ ^29^, ^30^ ^]^ Sole GelMA core was tightly aggregated due to micro‐scale adhesion forces, as shown in **Figure** **2**a. The microfiber was divided into 96 or more segments, each ≈1 cm in length. These segments were then transferred into corresponding wells of a 96‐well dish to create 96 skeletal muscle microPMs, as depicted in Figure 2b. After being cultured to form a myobundle in the core, the microPMs were transferred onto a tailor‐made force/electrical hybrid loading system for subsequent micromanipulation, as shown in Figure 2c and Figure S3 (Supporting Information). The two ends of the microPM could be directly placed on a two‐probe actuator, while its middle part was immersed in the culture medium with 1% w/v sodium citrate. The shell of the microPM was degraded, leaving the core stably suspended on the two‐probe actuator. The shell degraded completely in just 30 s after 4 days of culture. A stretching force (*F_s_*) was then applied along the long axis of the core, which was measured by the deflection of the sensing probe when the moving probe moved away from it (as shown in Figure 2d; Figure S4 and Video S1, Supporting Information). Additionally, we demonstrated that the shell‐involved transfer and degradation method was highly biocompatible for the myobundles fabricated in the cores with two cell densities, as shown in Figure 2e.

When the distance between two probes, *L_gap_*, was greater than 13 mm, a step increase phenomenon of *F_s_* was frequently observed due to the viscous hysteresis of the core. However, when *L_gap_* was limited to 7 mm, there was a continuous increase of *F_s_* with increasing *L_gap_* (as shown in Figure 2f). Therefore, we always keep initial *L_gap_* at ≈5 mm, and the stretched distance of the core is less than 30%. Additionally, a liquid bridge can provide an adhesion force of ≈120 µN to fix the core without the need for clamps. To prevent fixation failure, we implemented model predictive control (MPC) to regulate the moving distance of the moving probe: *L_m_* to control the resulting *F_s_* to track the reference force command: *F_cmd_* of less than 120 µN, as shown in Figure 2g. The MPC control process was explained in Figure S5 (Supporting Information). Figure 2h and Video S2 (Supporting Information) demonstrated a regular sinusoidal waveform of *F_s_* with a maximum value of 15 µN was generated at three different frequencies (0.01, 0.1, and 1 Hz) during *F_s_*‐controlled cyclic loading. However, without *F_s_*‐control, the waveform of *F_s_* was distorted and the maximum value of *F_s_* exceeded 15 µN, reaching ≈20 µN, which led to an inaccurate evaluation of viscoelasticity. The schematic of viscoelasticity measurement for the complex modulus E* and mechanical loss factor *tanδ* was shown in Figure 2I. Based on the *F_s_*‐control, the stress (*σ_s_*) and strain (*ε_s_*) of the core were calculated from *F_s_* and *L_m_*, and then transformed into the frequency domain using the Fast Fourier Transform (FFT) algorithm, allowing for obtaining their amplitudes (*A_ε_* & *A_σ_*) and phase angles (*ϕ_ε_* & *ϕ_σ_*) at the cell's mechanosensing frequency of 0.1 Hz.^[^ ^31^ ^]^ Finally, we determined the E^*^ and *tanδ* using Equation (7). Viscoelasticity calculation is also explained in the Supplementary materials of Section 12.

### 2.2 Hydrogel Microfiber To Cellular Microbundle Transition (HCT) in microPMs

**Figure 3:**
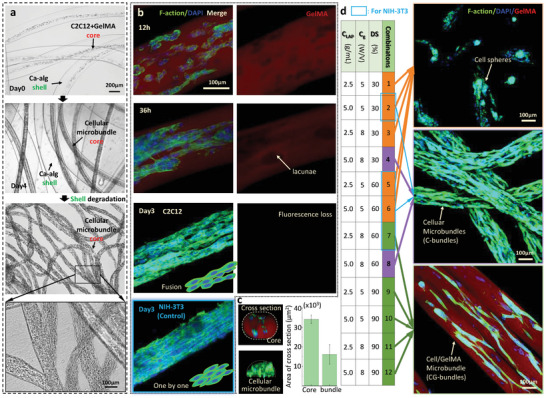
C‐bundle formation in GelMA core of microPMs. a) Transition process from C2C12‐laden GelMA core to metre‐long C‐bundle. b) Fluorescently labeled transition process for C2C12 and NIH‐3T3. c) Cross section of core and C‐bundle. Data are expressed as mean ± SD (n ≥ 50). d) Three cellular structures formed in 12 free combinations of three degrees of methacryloyl substitution (DS), two GelMA concentrations (C_g_), and two lithium acylphosphinate (C_LAP_) concentrations.

**Figure** **3**a illustrated the transition process of obtaining a myobundle from the core of C2C12‐laden GelMA after the shell had been degraded. Similarly, for microPMs, the same transition process was achieved with only 300 µL of culture media per well. We further used fluorescently labeled GelMA to demonstrate the correlation between cellular microbundles growth and GelMA core degradation, as shown in Figure 3b. During the first 12 h, hydrogel degradation created lacunae in the core, where cells were dispersed. Over the course of 36 h of culturing, the dispersed cells gradually connected with each other to form locally aligned cellular structures. Cell‐mediated GelMA degradation produced a locally hollowed core, while acellular areas in the core remained stable. Here, we verified that heterogeneity ensured the integration of cells with the core, leading to a successful transition and ultimately tissue growth. After three days of culturing, the core continued to degrade, resulting in fluorescence loss, and a bundle‐shaped C2C12 structure was formed. Subsequently, the growth medium was replaced with differentiation medium for myogenic differentiation. The whole process is defined as hydrogel microfiber to HCT in the following paper. The HCT can also be achieved by NIH‐3T3 fibroblasts as control groups. The difference in the resulting cellular microbundles between C2C12 and NIH‐3T3 can be distinguished by cell morphology after fluorescence staining. Primary C2C12 cell fusion was observed, whereas NIH‐3T3 cells were connected one by one. The cross‐section of the cell‐laden core was rounded rectangular, while the cross‐section of the cellular microbundle changed into a semi‐circle due to cell aggregation. The area of the core cross‐section was more consistent compared to the microbundles, as shown in Figure 3c and Figure S6 (Supporting Information).

We assessed the impact of various combinations of degree of methacryloyl substitution (DS), LAP concentration (C_LAP_), and GelMA concentration (C_g_) on HCT. C_g_ of 5%w/v represents the resulting molecular network of GelMA with low network density, which has been demonstrated to provide a suitable environment for 3D cell growth.^[^ ^32^ ^]^ Based on trial‐and‐error experiments, we found cell fusion might be inhibited when C_g_ was larger than 8% w/v regardless of DS and C_LAP._ Therefore, two representative C_g_ were chosen at 5% and 8% w/v, respectively. The increasing DS can lead to better mechanical performance of GelMA but reduce degradation rate. In general, DS was chosen at 30%, 60%, and 90% (±5%) to form three representative GelMA types with distinguishable material performance.^[^ ^30^ ^]^ For widening sample size to investigate the process of cell growth correlated with GelMA degradation, we further used two C_LAP_ to modify the degree of cross‐linking to influence the density of the molecular network. C_LAP_ of 0.5 mg mL^−1^ represents excessive photoinitiator according to the product manual, while C_LAP_ of 0.25 mg mL^−1^ represents normal photoinitiator.^[^ ^30^ ^]^ The results revealed the formation of three types of cell constructs: agglomerated cell spheres, cellular microbundles with or without GelMA residue, in 12 different combinations, as depicted in Figure 3d. The cell spheres indicated that the core degradation rate was faster than the rate of tissue formation. The rapid degradation of the core removed the scaffold for cell adhesion before the stable formation of cell–cell connections. As a result, C2C12 or NIH‐3T3 cells self‐aggregated to form spheres. The cellular microbundles with GelMA residue (CG‐bundle) indicate that the core degradation rate was slower than the rate of cell proliferation. The surrounding hydrogel network inhibits the space of cell growth and further decelerates core degradation to retain the integrated microfiber structure. For cellular microbundles without GelMA residue (C‐bundle), cells that are aligned tightly with each other and secrete ECM can replace the surrounding GelMA hydrogel to maintain the aligned arrangement after the shell degrades.

### 2.3 Micromanipulator‐Actuated Evaluation for Viscoelasticity and Contractile Force

**Figure 4:**
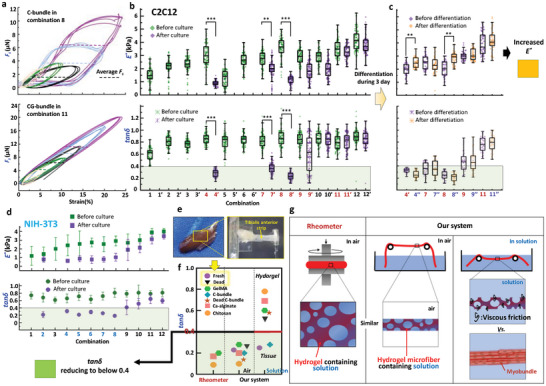
Viscoelasticity analysis for C‐bundles and CG‐bundles. a) *F_s_*‐strain curve of C‐bundels and CG‐bundles. b) Viscoelastic parameters, E* and tanδ, of the core before culture, C‐bundles and CG‐bundles after 3 days of culturing for C2C12. Data is presented as a box‐whisker plot, and n>80 for each combination. **p < 0.01, ***p < 0.001. c) E* and *tanδ* of myobundles and G‐myobundles after 3 days of differetation. n>30 for each combination. Establishment of the criteria of increased E^*^ evaluating *HCT*. Yellow blocks indicating the screening results satisfying the criteria. d) E* and tanδ, of the core before culture, C‐bundles and CG‐bundles after 3 days of culturing for NIH‐3T3. Data is presented as a box‐whisker plot, and n>80 for each combination. e) TA measurement. f) Comparison of *tanδ* between different hydrogels, engineered myobundles and artificial tissues. Establishment of the criteria of increased *tanδ* reducing to below 0.4 evaluating HCT. Green blocks indicating the screening results satisfying the criteria. g) Measurement comparison between the rheometer and our system.

E* and *tanδ* was measured under a strain amplitude of 10%, which was chosen based on the analysis of tensional homeostasis in myobundles during dynamic stretching (Figure S7, Supporting Information).^[^ ^33^ ^]^ **Figure** **4**a illustrates Lissajous figures that describe the relationship between *F_s_* and strain for C‐bundle and CG‐bundle in combinations of 8 and 11, respectively. When the strain is less than 15%, the softening of myobundles enables the average value of oscillating *F_s_* to remain almost constant despite a linear increase in C‐bundle length. In contrast, the average *F_s_* increases with the elongation of CG‐bundle. As shown in Figure 4b for C2C12 myoblasts, the E* was maintained within the range of 0.81–4.23 kPa to provide an extremely soft environment for cell growth. After 3 days of culturing, the *E* ^*^ of the core decreased, except for those from combinations 11 and 12, as well as the initially rejected combinations due to the formation of cell spheres (1, 2, 3, 5, and 6). The *tanδ* of all cores remained within the range of 0.45–1.1 before cell growth. After culturing, the *tanδ* of C‐bundles in combination 4 and 8, as well as some CG‐bundle samples in combinations 7 and 9, was distributed in a significantly narrower range of 0.1–0.4. However, the *tanδ* in combinations 10, 11, and 12 did not show significant differences. Additionally, the representative combination of 4, 7, 8, 9, and 11 was chosen to evaluate the effect of subsequent myogenic differentiation on E* and *tanδ*, as shown in the Figure 4c. The combinations of 4 and 8 represent the myobundles which are differentiated from C‐bundles, while the combinations of 7, 9, and 11 represent the G‐myobundles which are differentiated from CG‐bundles with different cell growth status. *E* ^*^ indicated the functional recovery of myobundles due to the distinguishable increase in its magnitude, while no obvious difference was observed in CG‐bundles before and after differentiation. More importantly, E^*^ could indicate the mechanical property of samples, and the increase of E^*^ was important reference for subsequent biofabrication. Previous results have directly demonstrated that the engineered 3D skeletal muscle in hydrogel could show an excellent capacity of supporting muscle regeneration in vivo, when it had a similar mechanical property to the tissue in vivo.^[^ ^34^ ^]^ Therefore, we set “increased E^*^ ” as a reference criteria for evaluating HCT. *tanδ* in these combinations could keep constant before and after differentiation, which indicated that cell differentiation had little effect on the connection strength between cells and between cells and hydrogel.

Figure 4d shows the measurement results of NIH‐3T3 fibroblast‐related E* and *tanδ* in 12 combinations used as control groups. Besides the combinations of 4 and 8, where both NIH‐3T3 and C2C12 cells could form pure cellular microbundles, NIH‐3T3 still showed stronger proliferation than C2C12, forming C‐bundles in the additional combinations of 2, 5, 6, and 7. The trend of E* and *tanδ* changed in NIH‐3T3 C‐ and CG‐bundles was the same as in C2C12, and the values of *tanδ* in all combinations forming C‐bundles were less than 0.4. Moreover, we compared our measured values of *tanδ* with ex vivo tibialis anterior strips (TA) from laboratory mice, as shown in Figure 4e. *tanδ* is ≈0.23 for fresh TA, while the *tanδ* of TA immersed in a 4% Paraformaldehyde fix solution for 2 h increased to ≈0.48, possibly due to the loss of cell function. A commercial rheometer (MCR102, Anton Paar, Austria) was employed to measure the *tanδ* of three hydrogels—GelMA, Ca‐alginate, and chitosan—to further validate our measured results (Figure 4f). The rheometer results showed that the *tanδ* of the three hydrogels was all below 0.4 and smaller than those measured by our system (0.5–0.9). We inferred that this measuring deviation could be attributed to the fact that the rheometer characterized hydrogels in air, whereas our system characterized hydrogel microfibers in solution. To verify this inference, we employed our system to characterize three hydrogel microfibers in air, and the results showed that all values were below 0.4, thus confirming our system's accuracy. Figure 4g elucidates the cause of the deviation for *tanδ*. For the rheometer, the measurement is achieved in air. Hydrogel containing solution has no connection with the surrounding air. Our system is similar to the rheometer when the measurement is conducted in air. However, when the measurement is conducted in solution, the solution contained within the hydrogel structure fully fuses with the solution surrounding the microfiber. This fusion induces a viscous friction between the microfiber and the surrounding solution when stretching‐induced relative movement occurs. Therefore, the viscous behavior of the microfiber is significantly exhibited, increasing the value of *tanδ* from ≈0.1 to greater than 0.5. When hydrogel microfibers are replaced with myobundles, the value of *tanδ* approximates that of real tissues (TA). Cells are independent of the surrounding solution; therefore, the effect of viscous friction can be neglected. Consequently, this deviation facilitates the criterion that “*tanδ* of less than 0.4” can serve as the key indicator for pure neotissue formation without hydrogel residuals.

**Figure 5:**
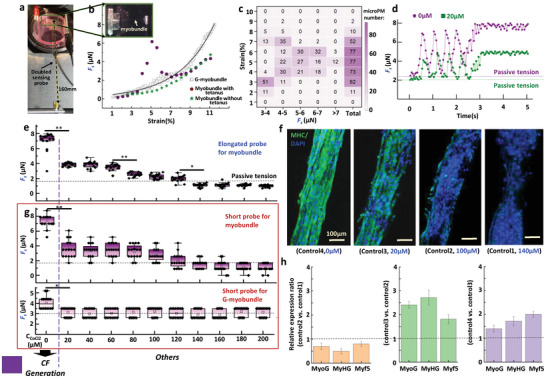
Contractile force (CF) analysis of myobundles. a) Two‐probe actuator with elongated sensing probe. b) Relationship between strain and *F_s_* for G‐myobundle, myobundle with and without tetanus force generation. c) Relationship between strain of myobundle and the number of samples generating tetanic force in 96 well. d) Representative *F_s_* (µN) response of myobundle successively stimulated with an electrical field at increasing frequencies (1, 5Hz) at C_cocl2_ of 0 and 20µM. e) Average *F_s_* (µN) response of myobundle in 11 CoCl_2_ concentration based on doubled sensing probe. Data is presented as a box‐whisker plot, and n=16 for each combination. *p < 0.05, **p < 0.01, ***p < 0.001. f) MHC and DAPI immunostaining images of myobundle at 4 representative CoCl_2_ concentration. g) Average *F_s_* (µN) response of myobundle for combination 8 and G‐myobundle for combination 9 in 11 CoCl_2_ concentration, which were measured by the previous short sensing probe. Based on measuring data, *F_s_* could indicate the CF generation that was utilized as the criteria to indicate the formation of myotubes. Purple block indicating the screening results satisfying the criteria. h) Comparison of gene expression levels of MyoG, MyHG, Myf5 in 4 representative CoCl_2_ concentration, normalized to GAPDH.

To enhance the sensitivity for the analysis of contractile behavior of micro‐scale myobundles, the length of the sensing probe was doubled, as shown in **Figure** **5**a. We first investigated the optimal length (*L_o_*) of myobundle to generate maximum tetanic CF based on the representative strain‐*F_s_* curve, as shown in Figure 5b. *F_s_* was increased exponentially as the strain on the G‐myobundle and myobundle increased. A sudden increase in *F_s_* was observed in the strain range between 4% and 6%, indicating the passive tension of the myobundle coupled with maximum active tension. Furthermore, the subsequent *F_s_* was larger than that of the G‐myobundle and myobundle without tetanic stimulation until the active force disappeared due to overlap between the actin and myosin filaments.^[^ ^35^ ^]^ In a random check of 96 microPMs, ≈77 models can generate CF in which the *L_o_* is mainly distributed in the strain range between 4% and 7% to produce a maximum *F_s_* of 8.32 µN, as shown in Figure 5c. Previous works show there is correlation between Cobalt Chloride (CoCl_2_) concentration and hypoxia level of C2C12 cells, and the use of CoCl_2_ increases hypoxia‐inducible factors 1 and 2, intracellular reactive oxygen species, and cell apoptossi in a dose‐dependent manner.^[^ ^36^, ^37^, ^38^ ^]^ Therefore, a CoCl_2_ concentration range of 0–200 µM was established to examine the effect of CoCl_2_‐induced hypoxia on the CF of myobundles. Our method allowed for high‐throughput culture and easy transferring manipulation, resulting in the establishment of 11 hypoxia conditions containing a total of 192 samples at one time. Under different hypoxia conditions, increased stimulation frequency fused the representative individual twitches of the myobundle into a stronger tetanic CF, as shown in Figure 5d. The twitches of the myobundle was shown in Video S3 (Supporting Information). Compared to active force, the *F_s_* measured at *L_0_* could provide a more distinct magnitude change due to the combination of active and passive tension. Therefore, *F_s_* at *L_0_* could be used for CF evaluation. In the subsequent paragraph, *F_s_* refers to the *F_s_* measured at *L_0_*.

Furthermore, we assessed the potential of CF as an indicator of myobundle injury. Based on an average of 16 samples for each concentration, we demonstrated that the *F_s_* of myobundle could generate a concentration‐dependent decrease. Four strengths of *F_s_* can be identified to evaluate the degree of damage to the myobundle (Figure 5e). The myotubes that are differentiated based on their morphology and expression of Myosin Heavy Chain (MHC) can be classified into four categories, corresponding to four strengths. This is shown in Figure 5f. In the case of myobundle, *F_s_* was smaller than the passive tension due to high concentration‐induced cell damage, which ultimately led to the collapse of the aligned cell structure and the formation of cell aggregates. However, this elongated probe was just utilized to confirm the availability of *F_s_* as indicator of CF. For achieving the measurement of E*, *tanδ* and *F_s_* for same one microMP, previous short probe continued to be used since the elongated probe was unsuitable for measuring viscoelasticity due to its fragility resulting from reduced flexural strength. The use of a short probe led to a change in the accuracy of *F_s_* measurement from 0.11 to 0.88 µN per pixel. Because *F_s_* was calculated by integral multiple of pixel, it was difficult to distinguish between two measured *F_s_* with a difference of less than 0.88 µN. Compared with four *F_s_* detected by the doubled probe, the short probe just had the capacity of clearly detecting two distinguished *F_s_* which could respectively indicate CF generation or not, as shown in Figure 5g. The similar behaviors also happened in G‐myobundles. Regarding gene expression, MyoG, MyHC, and Myf5 genes exhibited similar expression trends corresponding to the strongest *F_s_* (Figure 5h). Therefore, CF generation can be effectively recognized by *F_s_* measured by our system, which can be further taken as the key criteria to indicate the formation of myotubes after cell differentiation.

### 2.4 Screening Analysis of CoCl_2_ Concentration Threshold Inducing the Failure of Exosome‐Protected Muscle Fiber Repair

**Figure 6:**
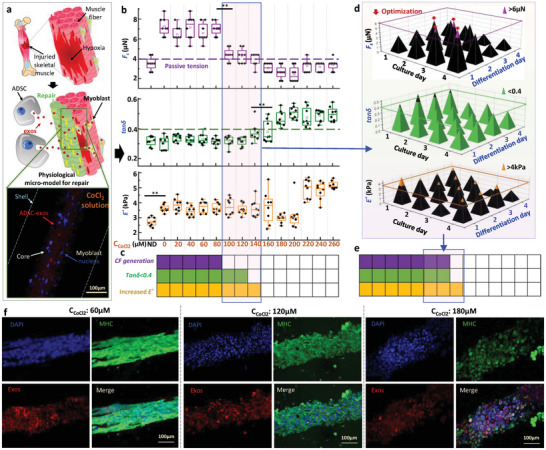
Screening analysis for the threshold of CoCl2 concentration inducing ADSC‐exos protection failure for muscle fiber repair in 3D. a) MicroPMs mimicking in vivo muscle fiber repair under hypoxia condition. b) E^*^, tanδ, and *F_s_* of 14 CoCl_2_‐induced hypoxia conditions. n=16 for each combination. c) Screening of the response of myobundle on 14 conditions based on three criterias. The highlighted grid indicates that the myobundle satisfies the corresponding criteria. d) Optimization of Culture period for C_CoCl2_ of 120 µM. e) Screening of the response of myobundle on 14 conditions based on three criterias after culture period optimization. The highlighted grid indicates that the myobundle satisfies the corresponding criteria. f) Immunofluorescent images about cell morphology with ADSC‐exos take‐up under three representative C_CoCl2_.

Muscle trauma or disease often induces a hypoxic condition that alters cell function in vivo. To repair damaged skeletal muscle, myoblasts fuse with injured muscle fibers. Additionally, exosomes secreted by mesenchymal stem cells (MSCs) can promote myoblast proliferation and differentiation in a hypoxic environment. However, the protective effect of exosomes on skeletal muscle remains to be elucidated.^[^ ^39^ ^]^ The core‐shell microfiber can be used as a physiological micro‐model to mimic such a repair process in vitro. The microfiber was created by encapsulating adipose‐derived mesenchymal stem cells (ADSC)‐derived exosomes (ADSC‐exos) with a concentration of 50 ug mL^−1^ and C2C12 myoblasts into the core. The microfiber was then subjected to a hypoxic environment by immersing it in media with CoCl_2_, as depicted in **Figure** **6**a. We established 14 hypoxia conditions dependent on CoCl_2_ concentration (C_CoCl2_) to screen for the threshold inducing failure of ADSC‐exos‐protected muscle fiber (MF) repair in 3D, and the response of *F_s_*, *tanδ* and E^*^ on C_cocl2_ was shown in Figure 6b. When C_CoCl2_ is less than 80 µM, the maximum passive tension increased to ≈4 µN, and the maximum *F_s_* reached ≈8 µN, indicating the effective protection of ADSC‐exos on MF repair. For C_CoCl2_ concentrations of 100, 120, and 140 µM, the *F_s_* of partial samples were slightly higher than *F_pmax_*, and almost all samples fell below *F_pmax_* at C_CoCl2_ concentrations of 160, 180, and 200 µM. The *tanδ* was less than 0.4 when C_CoCl2_ was less than 120 µM. Additionally, C_CoCl2_ at 140 µM was also classified as having a *tanδ* of larger than 0.4 since there was one sample with a *tanδ* greater than 0.4. Except for the increase induced by differentiation, there was no obvious difference in E* for the samples under C_CoCl2_ concentrations of less than 160 µM. However, when compared to C_CoCl2_ concentrations of less than 160 µM, the E* significantly decreased at concentrations of 180 and 200 µM before increasing again to a larger value. As shown in Figure 6c, if CF generation was used as the sole screening criteria, the protective function of ADSC‐exos had failed when C_CoCl2_ was greater than 80 µM. However, by combining with *tanδ*, we found that the HCT was achieved at C_CoCl2_ concentrations of 100 and 120 µM. Therefore, we inferred that culture or differentiation time could be further optimized to reduce exposure time in hypoxic conditions or provide enough time for myotube formation. We extended the previous 3+3 days culture period into 4 days of culture with 4 days of differentiation into 16 culture periods. We used E*, *tanδ*, and *F_s_* at C_CoCl2_ of 0 µM as a reference and successfully identified 4 optimized periods that showed exosome‐protected MF repair can be achieved at C_CoCl2_ of 120 µM, as shown in Figure 6d. Based on the screening data, we found that only one day of differentiation can facilitate myotube formation after four days of culturing. This is possibly due to the combination effect of mechanical stimulation provided by the shell and ADSC‐exos, which can promote myoblast proliferation and differentiation without the stimulation of horse serum.^[^ ^40^ ^]^ Similar screening results can also be achieved with C_CoCl2_ at a concentration of 100 µM, while it failed at a concentration of 140 µM, as shown in Figure 6e. Therefore, the threshold value was finalized at 120 µM, which can provide a novel insight into protecting against hypoxia‑induced muscle wasting. The correctness of the screening results was confirmed according to the representative fluorescent images of ADSC‐exos and differentiated myoblasts, as shown in Figure 6f. At a concentration of 60 µM C_CoCl2_, fused myoblasts were observed, and ADSC‐exos aggregated around cell nuclei instead of being uniformly distributed due to successful cellular uptake. Myoblasts that had fused returned to their individual state when exposed to C_CoCl2_ at a concentration of 120 µM. However, the transition from hydrogel to tissue was not fully achieved. At a concentration of 180 µM, apoptosis‐induced cell aggregation was clearly observed despite the formation of cellular uptake.

### 2.5 Screening Optimization of Composite Bioink for Muscle Tissue Regeneration

**Figure 7:**
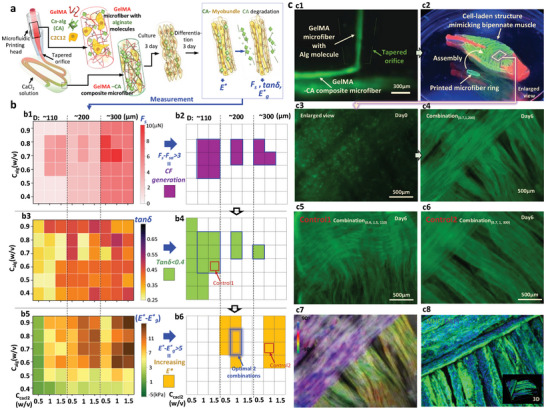
Screening optimization of composite ratio of Ca‐alginate (CA) in GelMA microfibers. a) Schematic of microfluidic spinning head to fabricate GelMA‐CA composite microfiber enabling aligned myoblasts growth into CA‐myobundle. b) CF, *tanδ* and E*‐based screening of CA‐myobundle for identifying optimized combination of alginate concentration, CaCl_2_ concentration and diameter of microfiber. (b1, b3, b5) Heat maps indicating original measurement data. (b2, b4, b6) Establishment condition of three screening criterias, and the corresponding screening results. c) Microfluidic bioprinting of bipennate muscle structure based on the optimized combination. (c1) Fluorescent image showing GelMA‐CA microfiber ejected from microfluidic printing head. (c2) Bioprinted cellular structures mimicking in vivo bipennate muscle. (c3) Enlarged view of local position of the cellular structures showing encapsulated C2C12 myoblasts in day 0. (c4) Cytoskeleton staining image showing the formation of myobundles. (c5) Control 1 showing distortion of assembled structure. (c6) Control 2 showing the lack of aligned cytoskeleton. (c7) Alignment direction of myobundes cytoskeleton in image of (c4), which was calculated by Image J. (c8) MHC and DAPI distribution in local position of assembled cellular structures.

Ca‐alginate hydrogel (CA) is frequently employed as a supportive element in the bioprinting of skeletal muscle, but lacks cell adhesion sites for cell spreading and proliferation. The proposed core‐shell configuration is not necessary for the fabrication of large‐volume muscle structure because CA shell prevents cells encapsulated different cores from connecting with each other. This study utilized the microfluidic device of Section 2.1 as a printing head for spinning GelMA‐CA composite microfiber bioink, as illustrated in **Figures** **7**a and S8 (Supporting Information). Within the glass tube of this head, GelMA microfiber was synthesized in alignment with the protocol outlined in Combination 8 of Section 2.2, before being compressed as it enters a CaCl_2_ solution via the head's tapered orifice. Owing to the presence of alginate molecules and myoblasts within the GelMA microfiber, the rapid formation of CA microfiber serves as a template, preserving the GelMA microfiber's compressed state and directing myoblast alignment. Although a more complex hydrogel structure is constructed in the composite microfiber relative to the pure GelMA core, the screening criteria derived from the core‐shell microfiber remain to be available for the ratio optimization of GelMA and CA to promote myobundle formation in the composite microfiber. Our extensive screening involved 432 samples across 54 combinations of alginate and CaCl_2_ concentrations, along with three microfiber diameters, as shown in Figure 7b. After three days of culturing and another three days of differentiation, C2C12 cells were able to proliferate and merge to form myobundles with CA (CA‐myobundles). The residual CA allows for the stable transfer of CA‐myobundles in air without a protective shell.

For *F_s_* measurement, CA in myobundle should be degraded due to its potential mechanical hindrance on the movement of contracting myotubes. It was noted that GelMA microfiber was formed prior to the CA template, resulting in all CA‐myobundles containing the same amount of GelMA hydrogel, regardless of the concentration of alginate and CaCl_2_. If there was a lack of effective cell growth and GelMA degradation to generate CF, *F_s_* mainly induced by the passive tension of GelMA hydrogel was nearly the same for CA‐myobundles with the same diameters, and the passive tension was increased with the increasing diameter of myobundles. When CF was generated, the resulting *F_s_* was significantly stronger than others with the same diameter (Figure 7 b1). Statistical analysis was used to subtract the average passive tension *F_sa_* of CA‐myobundles with the same diameters from the *F_s_*. The criteria for CF generation could be significantly satisfied when the difference is larger than ≈3 µN. Out of 54 combinations, 11 were screened (Figure 7 b2). If there is tissue formation matched with GelMA degradation, CA removal enables only pure cell and ECM structure to remain, which can be detected by the criteria of *tanδ* of less than 0.4 (Figure 7 b3). Two combinations, (0.8, 0.5, 300) and (0.7, 1, 300), were excluded based on their *tanδ* values of 0.75 and 0.718, respectively. Nine combinations were further screened out of the 11 initially considered (Figure 7 b4). A high concentration of CA component could alleviate the cell contractility‐induced distortion of myobundles. The CA residue is important to ensure the self‐folding of assembled structures during long‐term culture after the CA‐myobundle has been assembled into a larger‐scale biomimetic structure. Finally, the difference between E* and E*_g_ indicates a significant change in the CA component of the CA‐myobundle before and after CA degradation (Figure 7 b5). E* and E*_g_ represents the complex modulus of CA‐myobundle before and after CA degradation, respectively. The criteria for identifying an increase in E* is a difference larger than 5 kPa based on statistical analysis. Only two combinations, combination (0.7, 1, 200) and (0.8, 1, 200), were selected for subsequent bioprinting (Figure 7 b6).

Moreover, a microfluidic printing system was employed to create a scaffold mimicking bipennate muscle structure, as depicted in Figure 7 c1,c2 and Video S4 (Supporting Information). The schematic of fabrication process was shown in Figure S9 (Supporting Information). To corroborate the screening outcomes, representative combinations were selected for microfiber spinning. The combination (0.7, 1, 200) exemplified optimized parameters, whereas combinations (0.6, 1.5, 110) and (0.7, 1, 300) served as control 1 and 2 for failing to meet the criteria for increased E* and a *tanδ* of less than 0.4, respectively. Examination of a scaffold's enlarged section revealed encapsulated myoblasts with a dispersed arrangement, as shown in Figure 7 c3. Following a combined 3+3 days of culture, multi‐myobundles became distinctly visible (Figure 7 c4). As a contrast, myobundles were evident in the combination (0.6, 1.5, 110); however, their distribution appeared more irregular compared to that in combination (0.7, 1, 200), due to insufficient mechanical properties for sustaining the stability of assembled microfibers (Figure 7 c5). In the combination (0.7, 1, 300), while the assembly structure was maintained stably, myobundles were not as clearly discernible (Figure 7 c6). Additionally, quantitative analysis of myobundle skeleton alignment and HMC distribution underscored the anisotropic organization of muscle tissues regenerated within the bioprinted scaffolds using optimally processed microfiber bioink, as shown in Figure 7 c7,c8.

## 3 Discussion

**Figure 8:**
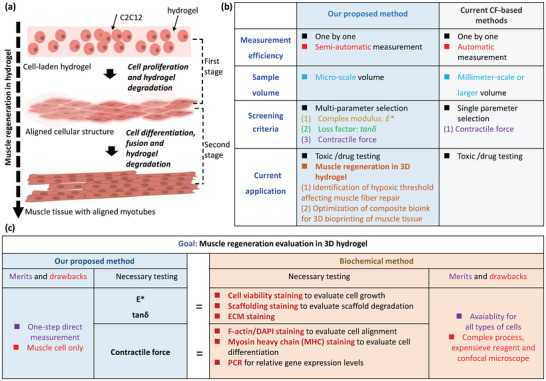
Illustration for the mechanism of our proposed method. a) Schematic diagram showing viscoelasticity and CF‐combined evaluation for the whole process of skeletal muscle regeneration. b) Comparison between our method and current CF assessment methods. c) Comparison between our method and traditional biochemical analysis.

Muscle regeneration within a 3D hydrogel occurs in two phases, as depicted in **Figure** **8**a. The initial phase involves the transformation of cell‐laden hydrogel into aligned cellular structures, followed by the differentiation and fusion of these aligned cells to create muscle tissue containing aligned myotubes. Although CF evaluation can confirm the presence of myotube formation, it struggles to assess the presence of hydrogel residue, the mechanical properties of the forming tissue, cell proliferation, and ECM secretion in 3D, among other factors. It is important to highlight that our proposed method incorporates the viscoelasticity parameter of E^*^ and *tanδ* into CF assessment to accurately evaluate hydrogel microfiber to HCT, focusing not merely on the contractile functionality of myotubes developed within hydrogel.^[^ ^41^ ^]^ It is a new finding that the HCT process can be reflected by viscoelasticity change of the microfiber‐shaped samples when characterized in solution rather than in air, which is correlated with the viscous friction between the microfibers with high‐aspect‐ratio, micro‐scale and porous structures and the surrounding solution. After analyzing a large dataset, we establish the following screening criteria:

1. Relying exclusively on CF generation for screening confirms the presence of muscle tissue with myotubes, but it may not exclude the presence of residual hydrogel, such as G‐myobundle in combination 9.
2. Evaluating both CF generation and *tanδ* of less than 0.4 in solution allows for the identification of pure muscle tissue, free from hydrogel residues, such as Myobundle in combination 8.
3. Integrating CF generation, *tanδ* of less than 0.4, and increased E^*^ into the evaluation process facilitates the creation of macro‐sized muscle tissue with enhanced mechanical properties, essential for the development of self‐folding structures, such as composite microfiber in combination (0.7,1,200).

In this paper, we have employed the term “high throughput” to describe our sample preparation process based on 96‐well plates. Additionally, we have used the term “large‐scale” to describe our screening method due to its ability to handle large amounts of experimental data. Our samples can be easily and quickly clamped and released from two probes without the need for chemical binders or complex manipulations during characterization, benefiting from the liquid bridge‐based fixation. It takes ≈2 min to place and test a single sample, and a total of ≈3 h for all 96 samples. Measuring the CF of engineered muscle tissues to evaluate their functionality has gained increasing interest. Popular methods currently used include microphysiological systems with microposts and biohybrid robots.^[^ ^42^, ^43^ ^]^ Figure 8b presents a comparison between our method and current CF‐assessment methods from four aspects:

1. Measurement efficiency: Due to weak CF, which causes only minor deformation of microposts or the mechanical structure of robots, a microscope is necessary to measure such deformations, or alternatively, a macro‐scale robot must be designed to produce a measurable movement distance. To improve the actuation of muscle bundles, a novel approach involving the integration of hyaluronic acid‐modified gold nanoparticles into a muscle bundle‐based biohybrid robot has been developed.^[^ ^44^ ^]^ However, the observation of samples is limited to one at a time, either due to the microscope's limited field of view or because each macro‐scale robot is designed to correspond with a single drug as a cost‐saving measure. In the current CF‐based evaluation method, samples must be examined individually. For our approach, samples must be manually placed onto two probes one at a time. As a result, achieving high‐throughput measurement with current methods poses a challenge.
2. Sample volume: The sample volume required by our method is significantly smaller than that of other methods, making large‐scale screening feasible. Our system allows easy manipulation of microfiber with dimensions of ≈10 mm in length and minimum 110 µm in diameter. Thus, this system requires only ≈0.01 mm^3^ in volume, and this is smaller than the volume of ≈0.05 mm^3^ used in the micropost‐based techniques and 0.5 mm^3^ in the force transducer‐based techniques, which can perform screening in a more economical way.^[^ ^10^, ^44^, ^45^ ^]^
3. Screening criteria: Three feature parameters of the sample can be provided at once for screening, while only the parameter of CF can be provided in other methods.
4. Current application: Our method benefits from a micro‐scale volume and a multi‐parameter selection mechanism. It can be applied not only to toxic/drug testing but also to the analysis of multi‐factor interactions on muscle regeneration in 3D. CF‐based methods currently only have the application of toxic/drug testing. Moreover, previous works demonstrated advanced and novel biofabricating methods for fabriating similar cellular structures proposed in Section 2.5, while they lacked an optimization method for synthesis parameters of hydrogel used as bioink.^[^ ^46^, ^47^ ^]^ Our screening method can achieve such optimization, so it should have great potential to attract lots of interesting in bioprinting and muscle tissue engineering.

Cells or tissues are traditionally characterized by immunofluorescence staining and quantitative PCR. These methods are destructive for cells and tissues,^[^ ^48^ ^]^ therefore, one sample usually responds to only one result. However, multiple processes of immunofluorescence staining and molecular biological technology are necessary for muscle tissue engineering. Our developed micromanipulation system evaluates myobundles through nondestructive mechanical testing, which allows the one‐step measurement of E*, *tanδ*, and CF for same one fiber‐shaped microPM. The multiple processes in the destructive method can be simplified by our proposed method. For instance, *tanδ* can replace the need for cell viability staining and scaffold staining to evaluate cell growth and scaffold degradation. The CF can replace the process of F‐actin/DAPI staining and MHC staining for evaluating cell alignment and differentiation in muscle tissue engineering. Figure 8c compares this method with biochemical methods, including immunofluorescence staining and molecular biological technology. However, the advantage of this method is just limited to muscle tissue engineering. Our proposed method can also process primary human myogenic cells or induced pluripotent stem cells into myobundles due to the high biocompatibility and easy processability of GelMA hydrogel.^[^ ^49^ ^]^ These human skeletal muscle physiological micro‐models have the potential to overcome the limitations of classical drug discovery‐to‐clinic pipelines. Moreover, for more biomimetic models of complete muscle regeneration, the presence of additional cell types such as macrophages, fibroblasts, and fibroadipogenic progenitor cells is required et al.^[^ ^50^ ^]^ Our proposed microfluidic spun method can be upgraded to enable multiple cell types to be assembled with complex spatial distribution and composition into one microfiber. In vitro skeletal muscle innervation can also be established by fabricating myobundles encapsulated by an outer nerve cell layer or nerve bundles embedded in a muscle cell layer.^[^ ^51^ ^]^ The proposed screening criteria also have great potential to be applicable to the aforementioned muscle cell types and other hydrogel systems characterized by our method. Because soft tissues are viscoelastic (*tanδ* from 0.1 to 0.2),^[^ ^18^ ^]^ “*tanδ* of less than 0.4” is versatile as long as other cells can grow and fuse into the corresponding tissue without hydrogel residues. However, *tanδ* values of common hydrogels characterized by commercial rheometers are below 0.4, such as GelMA (0.01‐0.4),^[^ ^52^ ^]^ alginate (0.16‐0.26),^[^ ^53^ ^]^ and chitosan (<0.1).^[^ ^54^ ^]^ The *tanδ* values of hydrogel microfibers evaluated in our system are higher than 0.4, which is mainly due to the characterization being carried out in solution rather than in air using a rheometer. At the microscale, the viscous friction between the porous scaffolds and the surrounding solution increased the viscous behavior of the microfibers, rendering viscoelasticity one of potential criteria for the screening of degraded hydrogel in our system. This phenomenon has been verified in the three representative hydrogels mentioned for tissue regeneration. Significantly, the *tanδ* of engineered myobundles and real tissue can increase to greater than 0.5 after cell death, as the myobundles and tissue can be approximated as ECM‐dominated hydrogels post‐cell death.^[^ ^55^ ^]^ Moreover, the solution is crucial for screening purposes within our system. On one hand, the presence of the solution maintains the homeostasis of the cells within the engineered microtissues; on the other hand, the solution facilitates the transmission of electrical stimulation to myobundles during contractile force measurement.

## 4 Conclusion

In this paper, we successfully demonstrated the feasibility of large‐scale screening using our proposed system with ≈3342 samples. We utilized viscoelasticity and contractile force as combinatorial screening criteria to provide comparable cues for screening skeletal muscle regeneration conditions. Our proposed two‐probe actuator under an electrical field offers the advantage of easy micromanipulation for high throughput. Compared to the traditional biochemical screening method based on the status of cultured cells, our new biophysical screening directly characterizes the physiological parameters of SMT in solution, taking into account cell organization and functionality. This approach can improve the accuracy of evaluating the effects of multifactorial interactions on skeletal tissue regeneration. Therefore, our proposed method provides a new approach to studying the complexity of various factors that contribute to the development and maintenance of native and artificial tissues.

## 5 Experimental Section

### System Configuration

A sensing and a moving probe were used to realize the viscoelastic characterization of microfiber along its long axis direction in the system. The moving probe (quartz, outer diameter (OD): 1 mm; inner diameter (ID): 0.6 mm) was installed at a motorized manipulator with 3 stepper motors (NSA12, minimum incremental movement was 0.2 µm), while the sensing probe (quartz, OD: 0.3 mm; ID: 0.1 mm) was installed at a manual manipulator. The bending deformation of the sensing probe was measured by a top‐view digital camera (DP21, frame rate: 5 Hz), then this bent deformation was used to calculate the force *F_s_* exerted on the microfiber by the program developed in LabView (version 2016). In addition, a laser micrometer (IG‐028) was utilized for a real‐time adjustment to ensure that two probes remain in the same horizontal plane. The overview of the system is shown in the Figure S3 (Supporting Information).

The characterization of the liquid bridge based fixation method is shown in Figure S10 (Supporting Information). To ensure the stable fixation, the MPC‐based *F_s_* control method was designed, the block diagram and design details can be referred from the Supplementary materials and Figure S5 (Supporting Information).

### Viscoelasticity Evaluation of microPM

The measurement of *F_s_* was based on the deflection of the sensing probe, which can be modelled using the Euler–Bernoulli beam theory, as demonstrated in our prior research.^[^ ^56^ ^]^ The parameters of the sensing probe are detailed in the Figure S4 (Supporting Information). Using these parameters, the relationship between the applied stretching force *F_s_* and the corresponding deflection of the sensing probe can be modelled as follows:

(1) $$\;\frac{y{\left(P\right)}^{\prime \prime }}{\left(1+y{\left(P\right)}^{\prime }\right)}=\frac{F_{s}l}{EI_{z}}$$

In which, *y*(*P*) is the deflection of the sensing probe in the position *P* during cyclic stretching, *l* is the fixed position of skeletal muscle physiological micro‐model (microPM) in the sensing probe which was smaller than the *L_p_*, *E* is elastic modulus of the quartz, *I_z_* is the moment of inertia of the cross‐sectional area:

(2) $$\;I_{z}=\frac{\pi }{64}\;\left(D_{p}^{4}-d_{p}^{4}\right)$$

In which, *D_p_* and *d_p_* were the outer and inner diameter of the sensing probe, respectively. However, due to the limited visual field of view, the sensing probe's tip deflection (*y*(*L_p_*) = *x*) was used to calculate the *F_s_* under the microscope. The boundary condition *y*(0) = 0 and *y’*(0) = 0 were combined with the value of *P* set as *L_p_*, given as:

(3) $$x=y\;\left(L_{p}\right)=-\frac{F_{s}l}{6EI_{z}}\left(3L_{p}-l\right)$$

As the values of the constants *l*, *E*, and *I_z_* can be obtained after placing the microPM on the sensing probe, the spring stiffness of the sensing probe (*k*) is defined as:

(4) $$k≔\frac{F_{s}}{x}\;=\frac{6EI_{z}}{l^{2}\left(l-3L_{p}\right)}\;$$

Thus, *k* is also a constant. According to the Equation (4), the *F_s_* can be calculated from the value of *x*. The *x* is measured using the Harris corner detection method on microscopic images. The calibration of the sensing probe and the microscopic camera is shown in Figure S11 (Supporting Information).

Next, the complex modulus E* and the mechanical loss factor *tanδ* were utilized to characterize the viscoelasticity of the microPM. To obtain these two parameters of the microPM, the imposed force of the microPM, *F_s_* is controlled to follow a signal *F_cmd_* with frequency *f_m_* = 0.1 Hz as showin in Figure 2g. The peak to peak value of the signal was limited to be less than 120 µN to satisfy the small amplitude oscillation. The imposed stress *σ_s_* is calculated as follows:

(5) $$\;\sigma_{s}=\frac{4F_{s}}{\pi d_{0}^{2}}\;$$

where *d_0_* is the initial diameter of the cell‐laden GelMA core. The corresponding strain *ε_s_* can be obtained as:

(6) $$\;\varepsilon_{s}=\frac{L_{m}-x}{L_{0}}\;$$

in which, *L_m_* is the moving distance of the moving probe that can be obtained from the motorized manipulator, *x* is the deflection of the sensing probe, and *L_0_* is the initial length of the core.

Based on the evaluated *ε_s_* and *σ_s_*, the FFT was applied to transform the *ε_s_* and *σ_s_* data from the time domain to the frequency domain, the *ε_m_* (*f*_m_) & *σ_m_* (*f*_m_). Therefore, their corresponding amplitude *A_ε_* (*f*_m_) & *A_σ_* (*f*_m_), and phase *ϕ_ε_* (*f*_m_) & *ϕ_σ_* (*f*_m_) in the frequency domain were calculated separately. Therefore, the value of E* and *tanδ* in the corresponding *f*_m_ can be further calculated as follows:

(7) $$\left\{\begin{array}{c} \;E^{*}=\frac{A_{\sigma }\left(f_{m}\right)}{A_{\varepsilon }\left(f_{m}\right)}\;\\ \tan\delta =\tan[\phi_{\sigma }\left(f_{m}\right)-\phi_{\varepsilon }\left(f_{m}\right)] \end{array}\right.$$

### Modulus Evaluation of microPM

The modulus of the microfiber was evaluated from the Lissajous‐Bowditch curves of the *σ_s_* and *ε_s_*. The final loop of the curves was choosed to calculated the modulus *E* as:

(8) $$E\;=\frac{\sigma_{la}-\sigma_{sm}}{\varepsilon_{la}-\varepsilon_{sm}}\;$$

where *σ_la_* and *σ_sm_* are the largest and the smallest stress of the loop, and *ε_la_* and *ε_sm_* are the largest and the smallest strain of the final loop in the curves, which are shown in Figure S2 (Supporting Information).

### Cell‐Laden Core‐Shell Microfiber Fabrication

The core‐shell microfiber were spun by a two‐layer coaxial nozzle that was used in our previous work.^[^ ^57^ ^]^

### Fluorescently Labeled GelMA

Fluorescently labeled GelMA were purhased from SuZhou Intelligent Manufacturing Research Institute (SuZhou, China).

### Immunofluorescene Staining and Imaging

To observe C2C12 morphology, F‐actin and cell nuclei were stained as follows: first, C2C12 encapsulated in microPMs were fixed in fresh, methanol‐free 4% formaldehyde in PBS at room temperature for 10 min and then washed three times in PBS with a 5 min incubation for each wash. Then, the fixed C2C12 were permeabilized with 0.1% Triton X‐100 in PBS for 10 min and rinsed again. Next, 6.6 µM Alexa Fluor 488 phalloidin was diluted at 1:20 in PBS, added to C2C12, incubated for 15 min at room temperature, then removed, and the sample was rinsed once with PBS. Fourth, the cell nuclei were stained with DAPI for a 10 min incubation.

For myotube immunostaining, the differentiated cell‐laden microfibers were fixed, permeabilized, and blocked as described above. Then, the microfibers were immersed in primary mouse monoclonal antibody MY‐32 (M1570, Sigma) at a dilution of 1:400 in DPBS, and incubated at 4 °C overnight in dark. The next day, microfibers were washed with DPBS for three times, then treated with goat anti‐mouse Alexa Fluor 488 antibody (ab‐150113, Abcam, Japan) at a dilution of 1:500 in DPBS, and incubated at room temperature in dark for 1 h. The samples were then stained with nuclei after washing with DPBS using 50 mg mL^−1^ DAPI for 15 min. The stained myotubes were washed gently and imaged with laser confocal microscopy

### MicroPM Responses under Electrical Stimulation

To observe the contractile activity of microPM, two parallel titanium plate electrodes were placed 5 cm apart on either side of the microPM. The microPM was placed between the two probes, and then stimulated with square waves with a pulse width of 10 ms, an electric field intensity of 5 V cm^−1^, and a frequency range of 1–5 Hz. The contraction of microPM was monitored, and the deflection of the sensing probe was recorded under the microscope. The contraction force was calculated using this recorded data.

### Tibialis Anterior Strip Extraction and Testing

The tibialis anterior strips were obtained from male Sprague‒Dawley rats (350–400 g, aged 10–12 weeks) sourced from the Chinese PLA General Hospital. All animal experiments were conducted in accordance with protocols approved by the aforementioned institution. The tibialis anterior tissue was thawed, and transferred to a petri dish containing fresh, relaxing solution. The tissue was secured with pins at both ends and submerged. The strip of tissue was then smoothly extracted along its longitudinal axis using forceps and transferred to a test petri dish with relaxing solution. Subsequently, the strip was placed between the two probes and for further testing.

### Fabrication of Bipennate Muscle Structure from Microfluidic Bioprinting

The microfluidic bioprinting and PDMS casting techniques were used to develop the bipennate muscle structure. Initially, a bipennate‐muscle‐shaped mold was created using PDMS mixed in a 1:10 ratio of Sylgard 184 and Sylgard 527. Subsequently, the resulting microfiber rings, labelled with green fluorescent, were produced using our developed microfluidic bioprinting system. Subsequently, the rings were carefully placed into the PDMS mold. The GelMA labelled with red fluorescent was used to fill the position of the muscle tendon. After exposure to UV light, the GelMA solidified. Finally, after pouring the GelMA solution without fluorescent and allowing it to solidify, the bipennate muscle structure canbe carefully removed from the PDMS mold. The fabrication processes were shown in the Figure S8 (Supporting Information).

### Coculture and Staining of ADSC‐exos and microPMs

The ADSC‐exos were diluted in culture medium and passed through a 0.22 µm filter to ensure sterility before the experiment. The ADSC‐exos were purified and labelled with the red fluorescence dye to examine their internalization in microPMs. A final concentration of 1 × 10^−6 ^M was achieved by incubating 250 µL of ADSC‐exos diluted in PBS with the dye for 5 min. The mixture was then ultracentrifuged for 70 min at 4 °C and 100 000 ×g to remove the supernatant. The resulting pellets were resuspended in PBS. MicroPMs were stimulated with fluorescent‐labelled exosome in serum‐depleted medium for 24 h. After fixation with 4% paraformaldehyde, cells were washed two times with PBS and the nuclei were counterstained with DAPI. The resulting images were observed under the microscope (Olympus, Tokyo, Japan).

### Statistical Analysis

One‐way analysis of variance (ANOVA) was used to statistically analyze the measured and scored data using GraphPad Prism 8 software (GraphPad Software, Boston, MA, USA). Results were presented as mean ± standard deviation (SD). The statistical significance was accepted at the *p*‐value < 0.05 (*p < 0.05 and **p < 0.01, ***p < 0.001 and ****p < 0.0001 versus control, ns = not significant).

## Conflict of Interest

The authors declare no conflict of interest.

## Supporting information

Supporting Information

Supplemental Video 1

Supplemental Video 2

Supplemental Video 3

Supplemental Video 4

## Data Availability

Data sharing is not applicable to this article as no new data were created or analyzed in this study.

## References

[1] S. Ostrovidov , S. Salehi , M. Costantini , K. Suthiwanich , M. Ebrahimi , R. B. Sadeghian , T. Fujie , X. Shi , S. Cannata , C. Gargioli , A. Tamayol , M. R. Dokmeci , G. Orive , W. Swieszkowski , A. Khademhosseini , Small 2019, 15, 1805530. 10.1002/smll.201805530 PMC6570559 31012262

[2] J. Wang , C. J. Zhou , A. Khodabukus , S. Tran , S.‐O. Han , A. L. Carlson , L. Madden , P. S. Kishnani , D. D. Koeberl , N. Bursac , Commun Biol 2021, 4, 524. 33953320 10.1038/s42003-021-02059-4 PMC8100136

[3] J. Fang , J. Sia , J. Soto , P. Wang , L. K. Li , Y.‐Y. Hsueh , R. Sun , K. F. Faull , J. G. Tidball , S. Li , Nat. Biomed. Eng. 2021, 5, 864. 33737730 10.1038/s41551-021-00696-y PMC8387336

[4] R. Mestre , N. García , T. Patiño , M. Guix , J. Fuentes , M. Valerio‐Santiago , N. Almiñana , S. Sánchez , Biofabrication 2021, 13, 045011. 10.1088/1758-5090/ac165b 34284359

[5] L. Yang , S. Pijuan‐Galito , H. S. Rho , A. S. Vasilevich , A. D. Eren , L. Ge , P. Habibović , M. R. Alexander , J. de Boer , A. Carlier , P. van Rijn , Q. Zhou , Chem. Rev. 2021, 121, 4561. 33705116 10.1021/acs.chemrev.0c00752 PMC8154331

[6] M. J. Mondrinos , F. Alisafaei , A. Y. Yi , H. Ahmadzadeh , I. Lee , C. Blundell , J. Seo , M. Osborn , T.‐J. Jeon , S. M. Kim , V. B. Shenoy , D. Huh , Sci. Adv. 2021, 7, eabe9446. 33712463 10.1126/sciadv.abe9446 PMC7954445

[7] L. Madden , M. Juhas , W. E. Kraus , G. A. Truskey , N. Bursac , Elife 2015, 4, e04885. 25575180 10.7554/eLife.04885 PMC4337710

[8] T. Nagashima , S. Hadiwidjaja , S. Ohsumi , A. Murata , T. Hisada , R. Kato , Y. Okada , H. Honda , K. Shimizu , Adv. Biosyst. 2020, 4, 2000121. 10.1002/adbi.202000121 33084245

[9] M. Guix , R. Mestre , T. Patiño , M. De Corato , J. Fuentes , G. Zarpellon , S. Sánchez , Sci. Rob. 2021, 6, eabe7577. 10.1126/scirobotics.abe7577 34043566

[10] M. Juhas , N. Abutaleb , J. T. Wang , J. Ye , Z. Shaikh , C. Sriworarat , Y. Qian , N. Bursac , Nat. Biomed. Eng. 2018, 2, 942. 30581652 10.1038/s41551-018-0290-2 PMC6296488

[11] M. Yadid , M. Hagel , M. B. Labro , B. L. Roi , C. Flaxer , E. Flaxer , A. R. Barnea , S. Tejman‐Yarden , E. Silberman , X. Li , R. Rauti , Y. Leichtmann‐Bardoogo , H. Yuan , B. M. Maoz , Adv. Sci. 2023, 10, 2207498. 10.1002/advs.202207498 PMC10520681 37485582

[12] M. Afshar Bakooshli , E. S. Lippmann , B. Mulcahy , N. Iyer , C. T. Nguyen , K. Tung , B. A. Stewart , H. van den Dorpel , T. Fuehrmann , M. Shoichet , A. Bigot , E. Pegoraro , H. Ahn , H. Ginsberg , M. Zhen , R. S. Ashton , P. M. Gilbert , Elife 2019, 8, e44530. 31084710 10.7554/eLife.44530 PMC6516829

[13] H. Vandenburgh , Tissue Eng., Part B 2010, 16, 55. 10.1089/ten.teb.2009.0445 PMC2865990 19728786

[14] C. Vesga‐Castro , J. Aldazabal , A. Vallejo‐Illarramendi , J. Paredes , Elife 2022, 11, e77204. 35604384 10.7554/eLife.77204 PMC9126583

[15] J. Gilbert‐Honick , S. R. Iyer , S. M. Somers , H. Takasuka , R. M. Lovering , K. R. Wagner , H.‐Q. Mao , W. L. Grayson , Biomaterials 2020, 255, 120154. 32562942 10.1016/j.biomaterials.2020.120154 PMC11192434

[16] P. A. Janmey , D. A. Fletcher , C. A. Reinhart‐King , Physiol Rev 2020, 100, 695. 31751165 10.1152/physrev.00013.2019 PMC7276923

[17] E. A. Aisenbrey , S. J. Bryant , J. Biomed. Mater. Res., Part A 2018, 106, 2344. 10.1002/jbm.a.36412 PMC6030485 29577606

[18] O. Chaudhuri , J. Cooper‐White , P. A. Janmey , D. J. Mooney , V. B. Shenoy , Nature 2020, 584, 535. 32848221 10.1038/s41586-020-2612-2 PMC7676152

[19] D. Huang , Y. Huang , Y. Xiao , X. Yang , H. Lin , G. Feng , X. Zhu , X. Zhang , Acta Biomater. 2019, 97, 74. 31400521 10.1016/j.actbio.2019.08.013

[20] Y. Ma , T. Han , Q. Yang , J. Wang , B. Feng , Y. Jia , Z. Wei , F. Xu , Adv. Funct. Mater. 2021, 31, 2100848.

[21] O. Chaudhuri , L. Gu , M. Darnell , D. Klumpers , S. A. Bencherif , J. C. Weaver , N. Huebsch , D. J. Mooney , Nat. Commun. 2015, 6, 6365. 10.1038/ncomms7365 PMC4518451 25695512

[22] A. Elosegui‐Artola , A. Gupta , A. J. Najibi , B. R. Seo , R. Garry , C. M. Tringides , I. de Lázaro , M. Darnell , W. Gu , Q. Zhou , D. A. Weitz , L. Mahadevan , D. J. Mooney , Nat. Mater. 2023, 22, 117. 36456871 10.1038/s41563-022-01400-4 PMC10332325

[23] E. E. Charrier , K. Pogoda , R. G. Wells , P. A. Janmey , Nat. Commun. 2018, 9, 449. 29386514 10.1038/s41467-018-02906-9 PMC5792430

[24] C. M. Madl , B. L. LeSavage , R. E. Dewi , C. B. Dinh , R. S. Stowers , M. Khariton , K. J. Lampe , D. Nguyen , O. Chaudhuri , A. Enejder , S. C. Heilshorn , Nat. Mater. 2017, 16, 1233. 29115291 10.1038/nmat5020 PMC5708569

[25] A. Elosegui‐Artola , Curr. Opin. Cell Biol. 2021, 72, 10. 33993058 10.1016/j.ceb.2021.04.002

[26] C. Liu , Q. Yu , Z. Yuan , Q. Guo , X. Liao , F. Han , T. Feng , G. Liu , R. Zhao , Z. Zhu , H. Mao , C. Zhu , B. Li , Bioact Mater 2023, 25, 445. 37056254 10.1016/j.bioactmat.2022.07.031 PMC10087107

[27] V. Ajmera , B. K. Kim , K. Yang , A. M. Majzoub , T. Nayfeh , N. Tamaki , N. Izumi , A. Nakajima , R. Idilman , M. Gumussoy , D. K. Oz , A. Erden , N. E. Quach , X. Tu , X. Zhang , M. Noureddin , A. M. Allen , R. Loomba , Gastroenterology 2022, 163, 1079. 35788349 10.1053/j.gastro.2022.06.073 PMC9509452

[28] Y. Tan , H. Huang , D. C. Ayers , J. Song , ACS Cent. Sci. 2018, 4, 971. 30159394 10.1021/acscentsci.8b00170 PMC6107872

[29] J. L. Drury , R. G. Dennis , D. J. Mooney , Biomaterials 2004, 25, 3187. 14980414 10.1016/j.biomaterials.2003.10.002

[30] J. He , Y. Sun , Q. Gao , C. He , K. Yao , T. Wang , M. Xie , K. Yu , J. Nie , Y. Chen , Y. He , Adv. Healthcare Mater. 2023, 12, 2300395. 10.1002/adhm.202300395 37115708

[31] B. Yang , K. Wei , C. Loebel , K. Zhang , Q. Feng , R. Li , S. H. D. Wong , X. Xu , C. Lau , X. Chen , P. Zhao , C. Yin , J. A. Burdick , Y. Wang , L. Bian , Nat. Commun. 2021, 12, 3514. 34112772 10.1038/s41467-021-23120-0 PMC8192531

[32] C. Colosi , S. R. Shin , V. Manoharan , S. Massa , M. Costantini , A. Barbetta , M. R. Dokmeci , M. Dentini , A. Khademhosseini , Adv. Mater. 2016, 28, 677. 26606883 10.1002/adma.201503310 PMC4804470

[33] D. Stamenović , M. L. Smith , Soft Matter 2020, 16, 6946. 32696799 10.1039/d0sm00763c

[34] P. M. Gilbert , K. L. Havenstrite , K. E. Magnusson , A. Sacco , N. A. Leonardi , P. Kraft , N. K. Nguyen , S. Thrun , M. P. Lutolf , H. M. Blau , Science 2010, 329, 1078. 20647425 10.1126/science.1191035 PMC2929271

[35] S. R. Devasahayam , (Ed.: S. R. Devasahayam ), Skeletal Muscle Contraction: Force and Movement. Signals and Systems in Biomedical Engineering: Physiological Systems Modeling and Signal Processing, Springer Singapore, Singapore 2019, 321.

[36] J. Muñoz‐Sánchez , M. E. Chánez‐Cárdenas , J. Appl. Toxicol. 2019, 39, 556. 30484873 10.1002/jat.3749

[37] T. L. Clanton , J. Appl. Physiol. 2007, 102, 2379. 17289907 10.1152/japplphysiol.01298.2006

[38] S. A. Ciafrè , F. Niola , E. Giorda , M. G. Farace , D. Caporossi , Free Radical Res. 2007, 41, 391. 17454121 10.1080/10715760601096799

[39] Z. Wang , J. Yang , X. Sun , X. Sun , G. Yang , X. e. Shi , J. Zhejiang Univ. Sci. B 2023, 24, 1. 36632747 10.1631/jzus.B2200243 PMC9837378

[40] W.‐W. Hu , Y.‐C. Chen , C.‐W. Tsao , S.‐L. Chen , C.‐Y. Tzeng , Bioeng. Transl. Med. 2024, 9, e10633. 38435819 10.1002/btm2.10633 PMC10905532

[41] F. J. Vernerey , S. Lalitha Sridhar , A. Muralidharan , S. J. Bryant , Chem. Rev. 2021, 121, 11085. 34473466 10.1021/acs.chemrev.1c00046

[42] M. E. Afshar , H. Y. Abraha , M. A. Bakooshli , S. Davoudi , N. Thavandiran , K. Tung , H. Ahn , H. J. Ginsberg , P. W. Zandstra , P. M. Gilbert , Sci. Rep. 2020, 10, 6918. 32332853 10.1038/s41598-020-62837-8 PMC7181829

[43] D. Kim , M. Shin , J.‐H. Choi , J.‐W. Choi , ACS Sens. 2022, 7, 740. 35138092 10.1021/acssensors.1c02125

[44] L. Wang , T. Li , Z. Wang , J. Hou , S. Liu , Q. Yang , L. Yu , W. Guo , Y. Wang , B. Guo , W. Huang , Y. Wu , Biomaterials 2022, 285, 121537. 35500394 10.1016/j.biomaterials.2022.121537

[45] A. D. Hofemeier , T. Limon , T. M. Muenker , B. Wallmeyer , A. Jurado , M. E. Afshar , M. Ebrahimi , R. Tsukanov , N. Oleksiievets , J. Enderlein , P. M. Gilbert , T. Betz , Elife 2021, 10, e60145. 33459593 10.7554/eLife.60145 PMC7906603

[46] A. Pardo , S. M. Bakht , M. Gomez‐Florit , R. Rial , R. F. Monteiro , S. P. B. Teixeira , P. Taboada , R. L. Reis , R. M. A. Domingues , M. E. Gomes , Adv. Funct. Mater. 2022, 32, 2208940.

[47] S. Choi , K. Y. Lee , S. L. Kim , L. A. MacQueen , H. Chang , J. F. Zimmerman , Q. Jin , M. M. Peters , H. A. M. Ardoña , X. Liu , A.‐C. Heiler , R. Gabardi , C. Richardson , W. T. Pu , A. R. Bausch , K. K. Parker , Nat. Mater. 2023, 22, 1039. 37500957 10.1038/s41563-023-01611-3 PMC10686196

[48] N. Sarkar , S. Bhumiratana , L. Geris , I. Papantoniou , W. L. Grayson , Nature Reviews Bioengineering 2023, 1, 361.

[49] M. Walker , P. Rizzuto , M. Godin , A. E. Pelling , Sci. Rep. 2020, 10, 7696. 32376876 10.1038/s41598-020-64725-7 PMC7203149

[50] J. Wang , A. Khodabukus , L. Rao , K. Vandusen , N. Abutaleb , N. Bursac , Biomaterials 2019, 221, 119416. 31419653 10.1016/j.biomaterials.2019.119416 PMC7041662

[51] H. Onoe , M. Kato‐Negishi , A. Itou , S. Takeuchi , Adv. Healthcare Mater. 2016, 5, 1104. 10.1002/adhm.201500903 26919482

[52] N. R. Barros , H.‐J. Kim , M. J. Gouidie , K. Lee , P. Bandaru , E. A. Banton , E. Sarikhani , W. Sun , S. Zhang , H.‐J. Cho , M. C. Hartel , S. Ostrovidov , S. Ahadian , S. M. Hussain , N. Ashammakhi , M. R. Dokmeci , R. D. Herculano , J. Lee , A. Khademhosseini , Biofabrication 2021, 13, 035030. 10.1088/1758-5090/aba503 32650324

[53] P. Duan , N. Kandemir , J. Wang , J. Chen , MRS Advances 2017, 2, 1309.

[54] P. Sánchez‐Cid , G. Gónzalez‐Ulloa , M. Alonso‐González , M. Jiménez‐Rosado , M. Rafii‐El‐Idrissi Benhnia , A. Romero , F. J. Ostos , V. M. Perez‐Puyana , Macromol. Mater. Eng. 2023, 308, 2300195.

[55] W. Kim , H. Lee , J. Lee , A. Atala , J. J. Yoo , S. J. Lee , G. H. Kim , Biomaterials 2020, 230, 119632. 31761486 10.1016/j.biomaterials.2019.119632 PMC7141931

[56] X. Chen , T. Sun , Z. Wei , Z. Chen , H. Wang , Q. Huang , T. Fukuda , Q. Shi , Biosens. Bioelectron. 2022, 214, 114517. 35803154 10.1016/j.bios.2022.114517

[57] T. Sun , Q. Shi , Q. Liang , Y. Yao , H. Wang , J. Sun , Q. Huang , T. Fukuda , Lab Chip 2020, 20, 3120. 32756693 10.1039/d0lc00544d

