# Supplementary material for: A Micromanipulation‐Actuated Large‐Scale Screening to Identify Optimized Microphysiological Model Parameters in Skeletal Muscle Regeneration
Source: Adv Sci (Weinh). 2024 Sep 12;11(44):2403622. doi: 10.1002/advs.202403622 (PMC11600204; doi:10.1002/advs.202403622)
Supplement: Supplementary file 1 — Supporting Information [file ADVS-11-2403622-s001.pdf]

ADVANCED SCIENCE
Open Access

Supporting Information

for *Adv. Sci.*, DOI 10.1002/advs.202403622

A Micromanipulation-Actuated Large-Scale Screening to Identify Optimized Microphysiological Model Parameters in Skeletal Muscle Regeneration

Xie Chen, Tao Sun, Shingo Shimoda, Huaping Wang, Qiang Huang, Toshio Fukuda and Qing Shi**

A Micromanipulation−Actuated Large−Scale Screening to Identify Optimized Microphysiological Model Parameters in Skeletal Muscle Regeneration

Xie Chen, Tao Sun, Shingo Shimoda, Huaping Wang, Qiang Huang, Toshio Fukuda and Qing Shi**

1. **Coaxial microfluidic spinning device**
2. **Modulus measurement**
3. **Overview of the micromanipulation system**
4. **F_s measurement**
5. **F_s-controlled method based on MPC**
6. **Cross-sections similarity evaluation**
7. **Analysis of tensional homeostasis**
8. **Microfluidic printing system**
9. **Fabrication processes of bipennate muscle**
10. **Measurement of maximum liquid bridge force**
11. **System calibration**
12. **Processes of viscoelasticity measurement**

1. Coaxial microfluidic spinning device

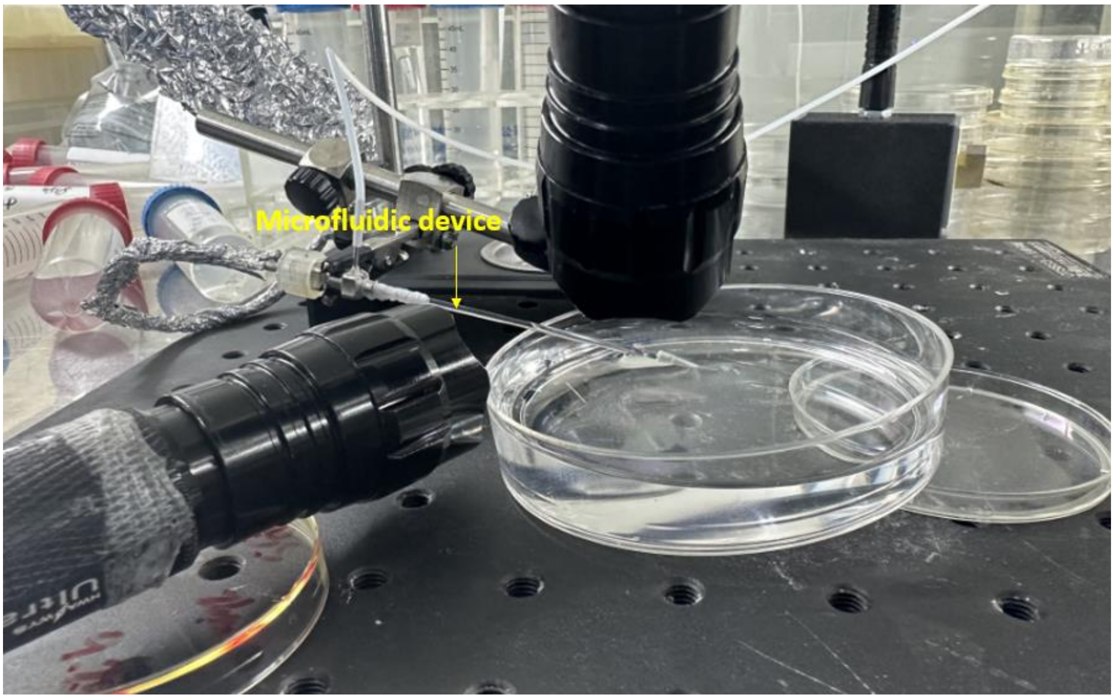

Figure S1. Coaxial microfluidic spinning device for the fabrication of core-shell microfibers.

2. Processes of viscoelasticity measurement

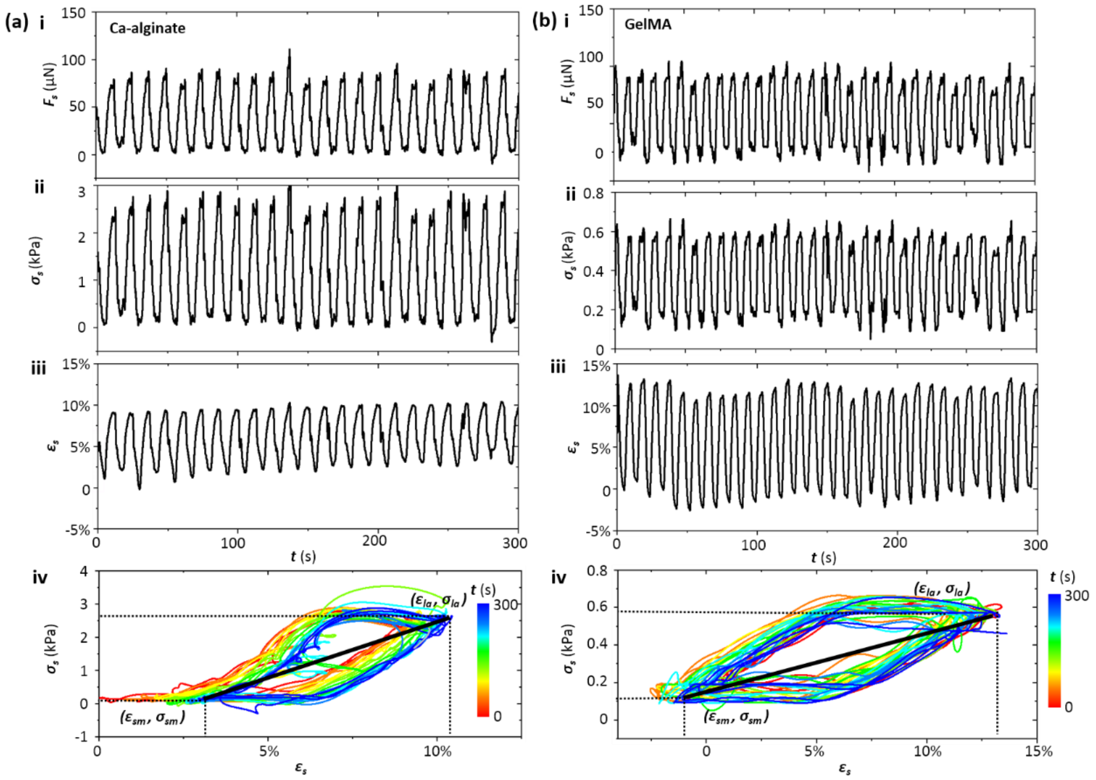

Figure S2. Elastic modulus of Ca-alginate (a) and GelMA (b) obtained from F_s-controlled cyclic stretching. (i) the applie force F_s, (ii) the stress σ_s, (iii) the corresponding strain ε_s, (iv) the resulting Lissajous-Bowditch curves during the cyclic stretching experiment of the microfiber.

3. Overview of the micromanipulation system

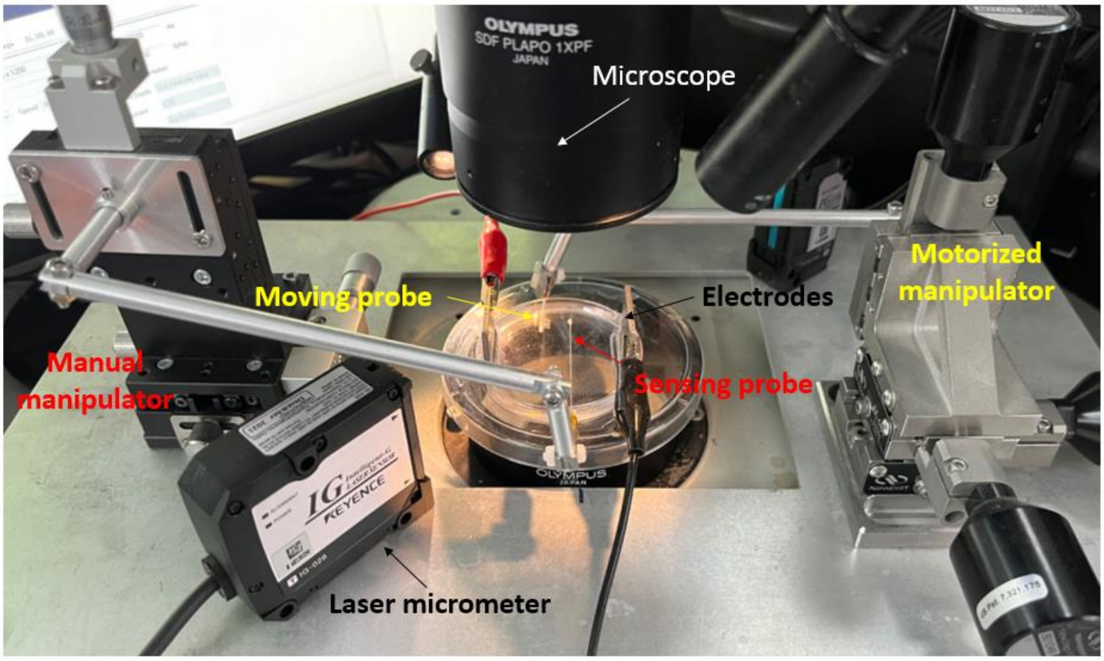

Figure S3. Overview of two-probe micromanipulation system with electrodes

4. F_s measurement

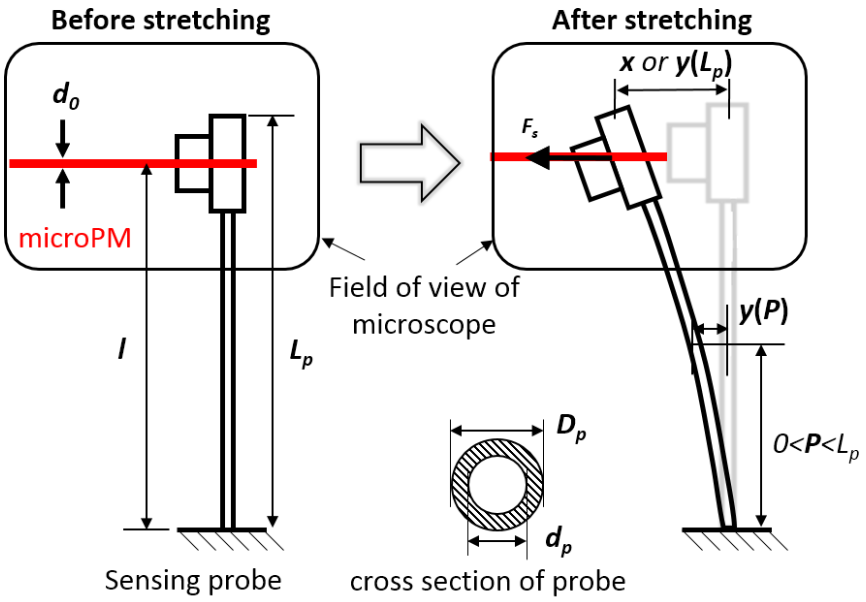

Figure S4. Structural parameters of the sensing probe

5. F_s-controlled method based on MPC

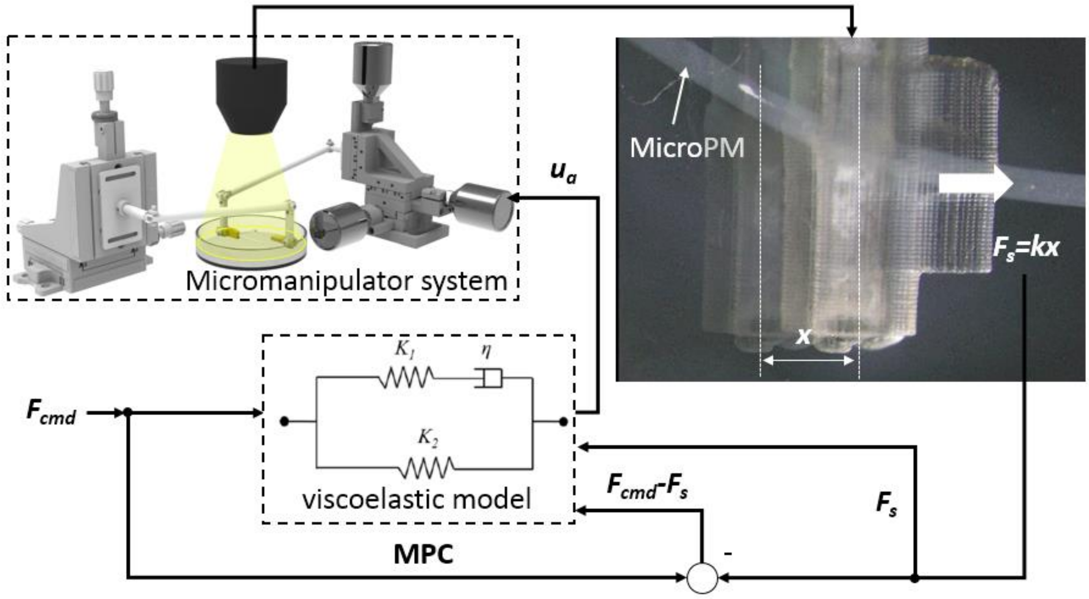

Figure S5. Block diagram of F_s control based on MPC

Table S1. Stress relaxation test and fitted values of microPM

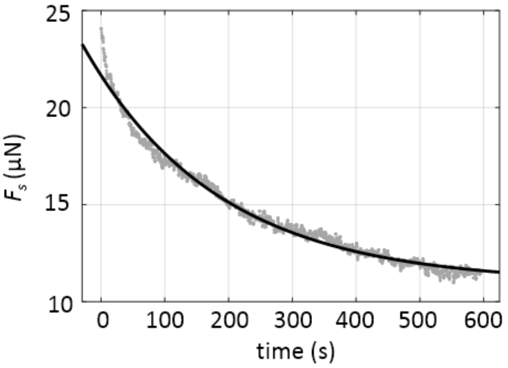

Fitted parameters	λ_1	λ_2	τ	ΔL (input)
Fitted value	10.70	10.97	211.9	1

The Model Predictive Control (MPC) was implemented to achieve the F_s control as shown in **Figure S5**. MPC is a control algorithm based on the mechanical model, so the mechanical model of the microPM was built based on its viscoelasticity, given as:

$$\frac{d\sigma_s}{dt}+\frac{K_1}{\eta}\sigma_s=(K_1+K_2)\frac{d\varepsilon}{dt}+\frac{K_1K_2}{\eta}\varepsilon_s \quad \text{(S1)}$$

in which, k_1, k_2, and η are the parameters of the microPM shown in **Figure S5**. σ_s, ε_s are the applied stress and corresponding strain of the microPM, which are calculated from equations (5) and (6) in the **Experimental Section/Methods.** To determine the exact value of the k_1, k_2, and η, we performed

the stress relaxation test. After performing some mathematical operations, the equation (S1) became:

$$F_s = (\underbrace{\frac{AK_2}{L_0}}_{\lambda_2} + \underbrace{\frac{AK_1}{L_0}}_{\lambda_1} e^{-t/\tau})\Delta L \tag{S2}$$

in which, L_0 and A are the initial length and area of the microPM. ΔL is the stretching distance of the microPM. The fitted values were listed as shown in **Table S1**.

Subsequently, the equation (S1) was written as following simplified state space model

$$\begin{cases} \dot{x}_p = -\frac{1}{\tau}x_p - \frac{AK_1}{L_0\tau}u_a = \underbrace{-\frac{1}{\tau}}_{a_p}x_p \underbrace{-\frac{\lambda_1}{\tau}}_{b_p}u_a \\ F_s = x_p + \frac{A(K_1+K_2)}{L_0}u_a = \underbrace{1}_{c_p}\bullet x_p + \underbrace{(\lambda_1+\lambda_2)}_{d_p}u_a \end{cases} \tag{S3}$$

in which, x_p is the state variable. The optimization problem was given as:

$$\min J(x_p, u_a, F_{cmd} - F_s) \tag{S4}$$

The control signal u_a of MPC can be solved from the equations (S3) and (S4) by using the interior point method, and the u_a can be used for F_s control.

6. Cross-sections similarity evaluation

Similarity	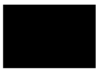	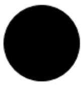	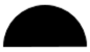	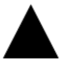
Binarization 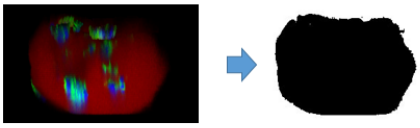	**0.8515**	**0.8674**	0.7798	0.7529
Binarization 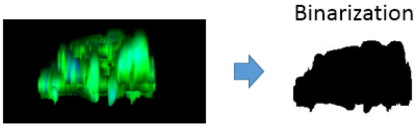	0.6472	0.7777	**0.9153**	0.7951

Figure S6. Cross-sections similarity evaluation based on contour matching algorithm

We employed the Euclidean distance-based contour matching algorithm to quantitatively characterize the cross-sections of the cell-laden core and myobundle. **Figure S6** presented the similarity of the cross-sections with other shapes. The images of the cell-laden core and myobundle were first binarized, and then four regular shapes were used for comparison and calculating the similarity. The results of the calculation indicated that the cell-laden core had the highest similarity when compared with a rectangle and circle, suggesting the cell-laden core was similar to a rounded rectangle. On the other hand, the myobundle received the highest similarity when compared with a semi-circle, indicating a closer resemblance to a semi-circle. Therefore, based on the binarized images, we concluded that the shape of the cell-laden core was a rounded rectangle, while the shape of the myobundle was closer to a semi-circle.

7. Analysis of tensional homeostasis

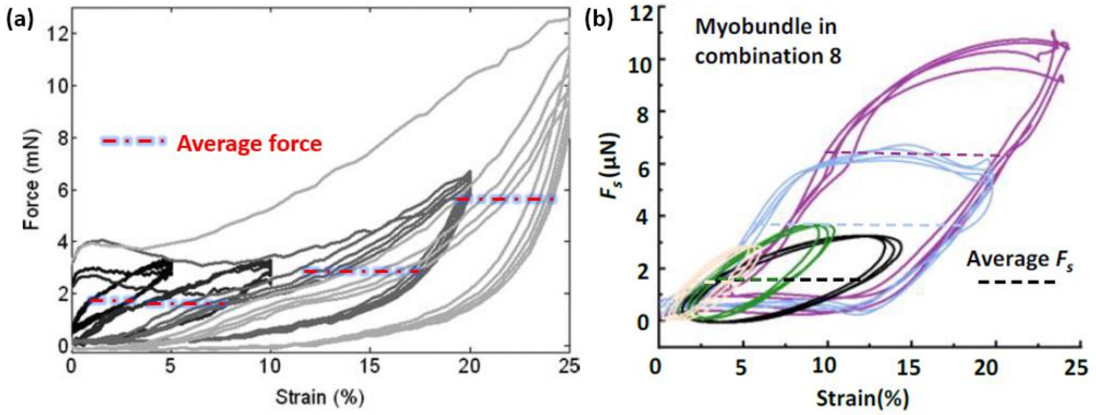

Figure S7. Comparison of force-strain curve during cyclic stretching. (a) cardiac fibroblast populated rings.[1] (b) myobundle in combination 8.

Tensional homeostasis is the ability of organs, tissues, and cells to maintain a homeostatic level of tension in response to external disturbances,[2] which can be easily observed in native tissues, such as tracheal smooth muscles. However, some studies have shown that when the degrees of deformation greater than 15%, the cells are destructed almost instantly and lead to the breakdown in the tensional homeostasis.[3-4] This breakdown in the tensional homeostasis can further make the average force to become non-constant,[1] as shown in **Figure S7 (a)**. **Figure S7 (b) (Figure 4c** in the manuscript) demonstrates that the average value of oscillating Fs remains constant despite a linear increase in myobundle length when the strain is less than 15%, suggesting tensional homeostasis. However, a nonlinear Fs-strain curve indicates that homeostasis cannot be maintained when the strain is larger than 15%. Thus, a strain value less than 15% has been selected for further experiments as a tradeoff between maintaining tensional homeostasis and producing detectable deformation.

8. Microfluidic printing system

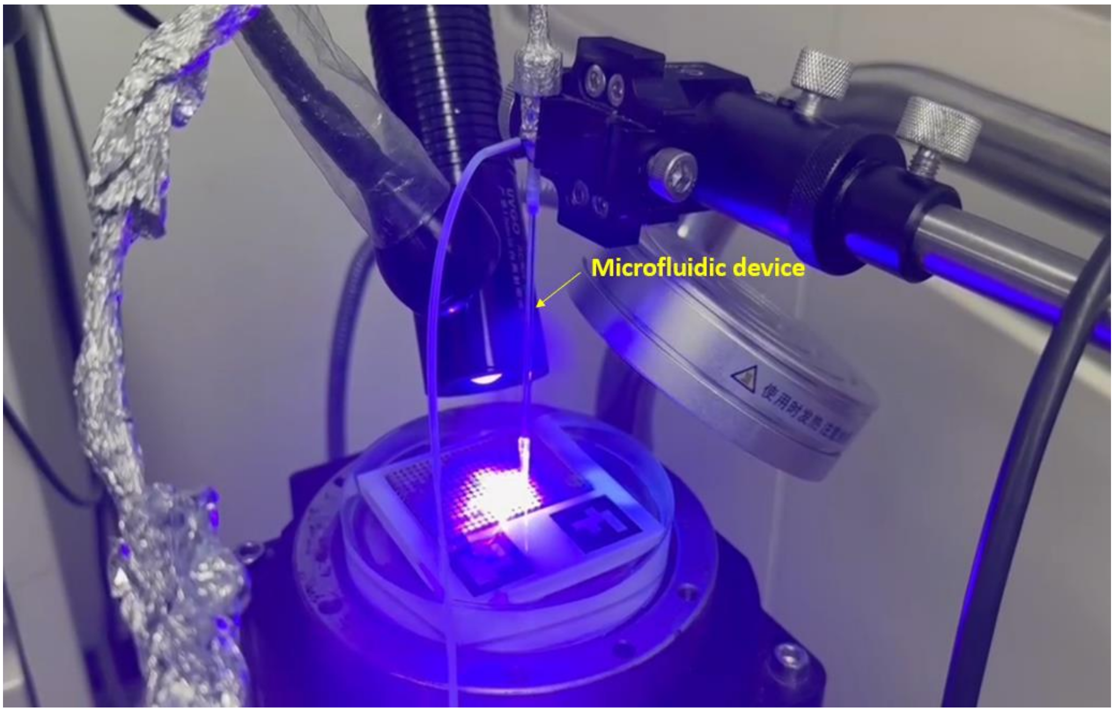

Figure S8. Microfluidic printing system for the fabrication of bipennate muscle structures

9. Fabrication processes of bipennate muscle

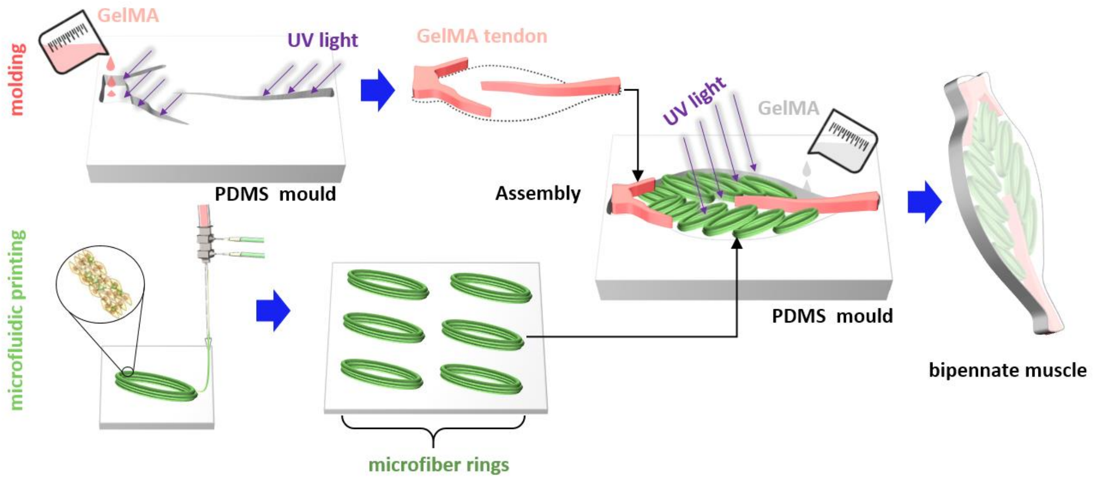

Figure S9. Fabrication processes of bipennate muscle

10. Measurement of maximum liquid bridge force

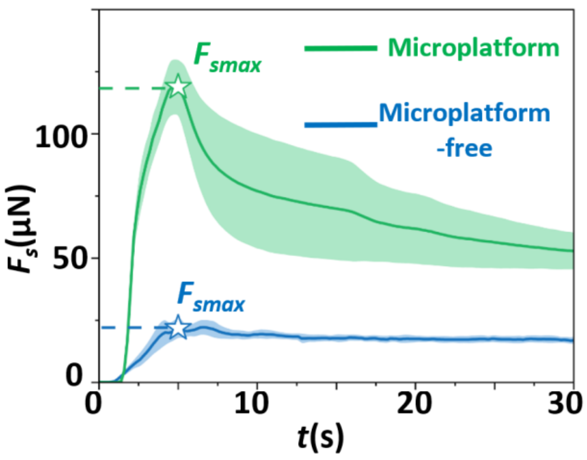

Figure S10. Comparison of F_s provided by microplatform and microplatform-free probes

The skeletal muscle physiological micro-model (microPM) can be placed on the surface of two probes, and a liquid bridge was simultaneously formed between the microPM and probe surface to stably fix the microPM. To improve the liquid bridge fixation force, a microplatform are mounted on the sensing probe and the moving probe. The microplatform are fabricated using Multijet Printing (MJP). After fabrication, the probes are inserted into the microplatforms, and they are fixed together with adhesive (Cyanoacrylate-based adhesive, Aronalpha, Inc.). By adding the microplatforms, the maximum F_s (~120 μN) was larger than the force (~20 μN) provided by microplatform-free probe to stably fix the micro-MP without any relative slide. **Figure S10** shows the time-dependent change of F_s with moving the moving probe away from the sensing probing.

11. System calibration

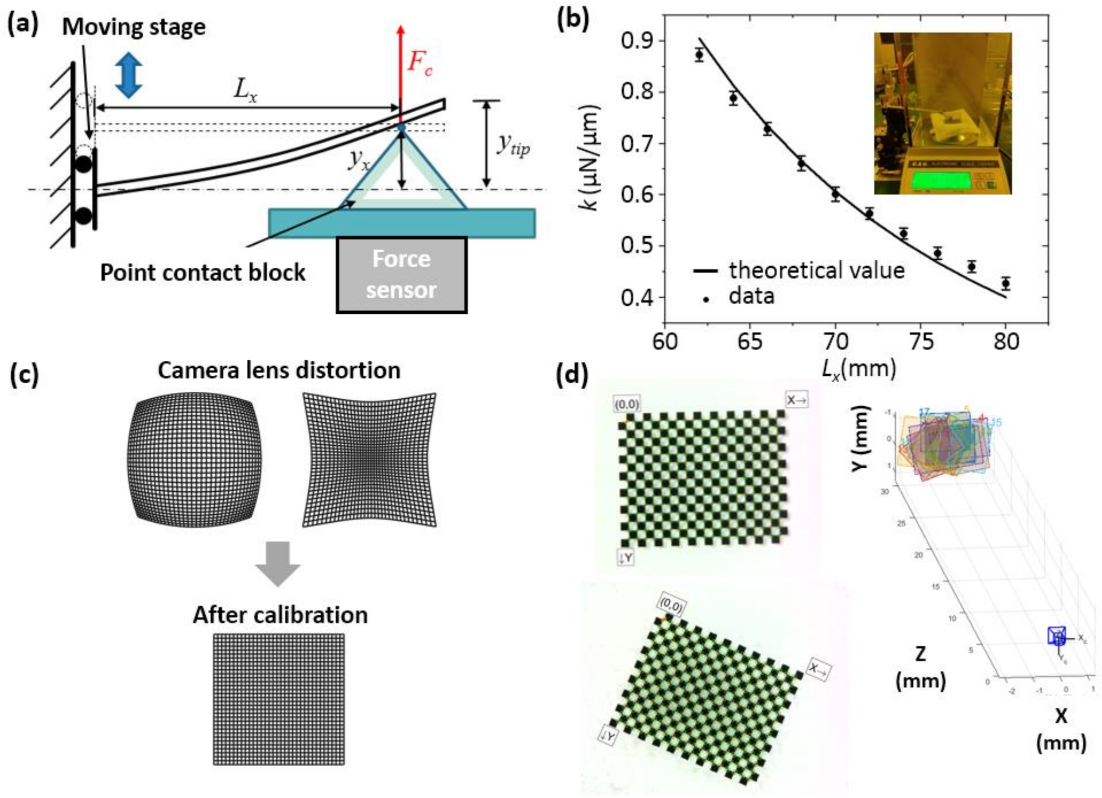

Figure S11. System calibration. (a) Schematic of the sensing probe calibration. (b) Calibration result of the sensing probe. (c) Schematic of microscopic camera lens distortion. (d) Microscopic camera calibration result.

To ensure accurate measurement of F_s, the spring stiffness of the sensing probe (k) should be validated according to equation (4) in the **Experimental Section/Methods** of the manuscript. The calibration of the sensing probe was inspired by the calibration processes of whiskers,[5] as shown in **Figure S11(a)**. The sensing probe was mounted in a precise moving stage. When the sensing probe moved downward and contacted with a point contact block, it began to bend. The corresponding force F_c can be read from the force sensor below by controlling the descent distance of the probe y_x. Thus, the spring stiffness of sensing probe in the contact position k_c can be obtained:

$$k_c = \frac{F_c}{y_x} \tag{S5}$$

However, the value of k_c is unequal to the value of k because the deformation at the contact position is unequal to the deformation at the tip of the sensing probe. Based on the equation (3) in the **Experimental Section/Methods** of the manuscript, the relationship between y_{tip} and y_x can be

modeled as:

$$y_{tip} = \frac{3L_p - L_x}{2L_x} y_x \tag{S6}$$

Thus, the k can be obtained as:

$$k = \frac{F_c}{y_{tip}} = \frac{2L_x F_c}{\left(3L_p - L_x\right) y_x} \tag{S7}$$

The calibration results were show in **Figure S11 (b)**. Considering the placement error due to the manual operation, the value of L_x is set as 79 mm and the resulting k is 0.4 μN/μm.

In addition, the microscopic camera should be also calibrated to compensate the camera lens distoration as shown in **Figure S11 (c)**. According to the Zhang's Camera Calibration Algorithm, the checkboard pictures were captured under the microscope and used for calibration,[6] as shown in **Figure S11 (d)**. After calibration, the average reprojection error was 0.08 which indicated that the camera distortion correction was completed. Therefore, the sensing probe's tip deflection x can be sensed at a resolution of 2.2 μm per pixel without distortion, and the F_s can be measured at a resolution of 2.2×0.4 = 0.88 μN/pixel.

12. Processes of viscoelasticity measurement

The scheme of viscoelasticity measurements was shown in **Figures 2g**, **h** and **i**. To measure the viscoelasticity of the microPM, several steps are implemented, as following:

1) The moving probe cyclically stretches microPM with a frequency of f_m under F_s-control. In this paper, the f_m is chosen at 0.1 Hz or subsequent measurements because the mechanosensing frequency of the cell is approximately 0.1 Hz.[7] The moving distance of the moving probe (L_m) can be directly read from the motorized manipulator, and the deflection of the sensing probe (x) can be obtained through image processing under the microscope.
2) The strain ε_s and stress σ_s of microPM are calculated using the L_m and x values, according to equations (S8) and (S9) below (equations (5)&(6) of the manuscript). Subsequently, the strain ε_s and stress σ_s data are transformed into the frequency domain using the Fast Fourier Transform algorithm (FFT). The corresponding amplitude $A_\varepsilon(f_m)$&$A_\sigma(f_m)$ and phase angle $\phi_\varepsilon(f_m)$&$\phi_\sigma(f_m)$ of the strain ε_s and stress σ_s can be directly obtained from the curve at the frequency 0.1 Hz, as the microPM is stretched under this frequency.

$$\sigma_s = \frac{4F_s}{\pi d_0^2} \quad \text{(S8)}$$

$$\varepsilon_s = \frac{L_m\text{-}x}{L_0} \quad \text{(S9)}$$

3) Finally, equation (S10) (equations (7) of the manuscript) can be used to evaluate the viscoelastic parameter of microMP including complex modulus E* and the loss factor from $A_\varepsilon(f_m)$&$A_\sigma(f_m)$ and $\phi_\varepsilon(f_m)$&$\phi_\sigma(f_m)$.

$$\begin{cases} E^* = \frac{A_\sigma(f_m)}{A_\varepsilon(f_m)} \\ tan\delta = tan[\emptyset_\sigma(f_m)\text{-}\emptyset_\varepsilon(f_m)] \end{cases} \quad \text{(S10)}$$

[1] J. J. Wille, E. L. Elson, R. J. Okamoto, Annals of Biomedical Engineering **2006**, 34 (11), 1678, https://doi.org/10.1007/s10439-006-9153-1.

[2] D. Stamenović, M. L. Smith, Soft Matter **2020**, 16 (30), 6946, https://doi.org/10.1039/D0SM00763C.

[3] K. D. Costa, W. J. Hucker, F. C.-P. Yin, Cell Motility **2002**, 52 (4), 266, https://doi.org/https://doi.org/10.1002/cm.10056.

[4] V. Panzetta, S. Fusco, P. A. Netti, Proceedings of the National Academy of Sciences of the United States of America **2019**, 116 (44), 22004, https://doi.org/10.1073/pnas.1904660116.

[5] W. Deer, P. E. I. Pounds, IEEE Robotics and Automation Letters **2019**, 4 (2), 1978, https://doi.org/10.1109/LRA.2019.2899215.

[6] Z. Zhang, IEEE Transactions on Pattern Analysis and Machine Intelligence **2000**, 22 (11), 1330, https://doi.org/10.1109/34.888718.

[7] B. Yang, K. Wei, C. Loebel, K. Zhang, Q. Feng, R. Li, S. H. D. Wong, X. Xu, C. Lau, X. Chen, P. Zhao, C. Yin, J. A. Burdick, Y. Wang, L. Bian, Nature Communications 2021, 12 (1), 3514, https://doi.org/10.1038/s41467-021-23120-0.
